# Lepidopteran defence droplets - a composite physical and chemical weapon against potential predators

**DOI:** 10.1038/srep22407

**Published:** 2016-03-04

**Authors:** Stefan Pentzold, Mika Zagrobelny, Bekzod Khakimov, Søren Balling Engelsen, Henrik Clausen, Bent Larsen Petersen, Jonas Borch, Birger Lindberg Møller, Søren Bak

**Affiliations:** 1 Plant Biochemistry Laboratory and Villum Research Center ‘Plant Plasticity’, Copenhagen Plant Science Center, Department of Plant and Environmental Sciences, University of Copenhagen, Copenhagen, Denmark; 2 Spectroscopy and Chemometrics, Department of Food Science, University of Copenhagen, Copenhagen, Denmark; 3 Copenhagen Center for Glycomics, Department of Cellular and Molecular Medicine , University of Copenhagen, Copenhagen, Denmark; 4 Plant Glycobiology, Department of Plant and Environmental Sciences, University of Copenhagen, Copenhagen, Denmark; 5 Department of Biochemistry and Molecular Biology, University of Southern Denmark, Odense, Denmark; 6 Carlsberg Laboratory, Copenhagen, Denmark

## Abstract

Insects often release noxious substances for their defence. Larvae of *Zygaena filipendulae* (Lepidoptera) secrete viscous and cyanogenic glucoside-containing droplets, whose effectiveness was associated with their physical and chemical properties. The droplets glued mandibles and legs of potential predators together and immobilised them. Droplets were characterised by a matrix of an aqueous solution of glycine-rich peptides (H-WG_11_-NH_2_) with significant amounts of proteins and glucose. Among the proteins, defensive proteins such as protease inhibitors, proteases and oxidases were abundant. The neurotoxin β-cyanoalanine was also found in the droplets. Despite the presence of cyanogenic glucosides, which release toxic hydrogen cyanide after hydrolysis by a specific β-glucosidase, the only β-glucosidase identified in the droplets (ZfBGD1) was inactive against cyanogenic glucosides. Accordingly, droplets did not release hydrogen cyanide, unless they were mixed with specific β-glucosidases present in the *Zygaena* haemolymph. Droplets secreted onto the cuticle hardened and formed sharp crystalline-like precipitates that may act as mandible abrasives to chewing predators. Hardening followed water evaporation and formation of antiparallel β-sheets of the peptide oligomers. Consequently, after mild irritation, *Zygaena* larvae deter predators by viscous and hardening droplets that contain defence proteins and β-cyanoalanine. After severe injury, droplets may mix with exuding haemolymph to release hydrogen cyanide.

Defensive secretions are widespread in animals and may be delivered to attackers by spraying, spitting, stinging, biting or smearing of the substances^1^. Defensive substances may consist of diverse components, such as secondary metabolites, peptides and proteins, which may have adverse effects on the physiological, locomotory, digestive or nervous system of predators^2^,^3^,^4^,^5^ and even pathogens^6^. Numerous insect species release or secrete such repellent, antinutritive or toxic compounds when attacked^7^,^8^,^9^ often via exocrine glands^10^. Some well-known examples of defensive secretions are the pygidial glands of bombardier beetles^11^, the glandular secretions of juvenile *Chrysomelina* leaf beetles^12^, the frontal glands of termite soldiers^13^ or urticating hairs of many lepidopteran larvae^14^. Thus, storage of toxins in special tissues and the immediate secretion of the toxin after physical irritation allow such insects to respond actively towards predator attacks.

A striking example of secretion-based defence in Lepidopterans has been reported in many species of the superfamily Zygaenoidea (e.g. burnets, foresters, slug moths)^15^. These larvae possess segmentally arranged cavities as part of their extraordinary thick cuticle^15^,^16^ (Fig. 1). Such cuticular cavities harbour highly viscous droplets known to contain cyanogenic glucosides (CNglcs)^16^,^17^. The defence droplets are released in response to mild physical irritation and can be reabsorbed when the irritation stops^16^. In aposematic *Zygaena filipendulae* larvae, droplet release is facilitated by contraction of segmental muscles around the irritated part of the body, which leads to an increased pressure of the haemolymph as well as within the cuticular cavity^16^. As a consequence, the cuticular cavity disrupts at its weakest part, i.e. the thin cuticular opening structure (Fig. 1), and a droplet is extruded^16^. Remarkably, neither specialised muscles nor specific cell types with morphological adaptations are involved in the secretion process, which renders such a defence system quite unique in comparison to other insect defence systems^16^: for example, *Chrysomelina* leaf beetle larvae possess specialised muscles connected to the secretory gland to control release of defensive secretion^18^, and the so-called easy bleeding by sawfly larvae relies on an integument with exceptional low mechanical resistance that ruptures under mechanical stress leading to haemolymph exudation^19^.

In *Z. filipendulae* larvae, defence droplets constitute the main site for accumulation of high concentrations of the two CNglcs linamarin and lotaustralin (~25 μg per μl)^17^, which are sequestered from the host plant^20^ and/or biosynthesised^21^. These CNglc-containing droplets may serve at least two functions in defence against predators: as a deterrent due to their bitter taste and as an active defence due to release of toxic hydrogen cyanide (HCN) by hydrolysing the CNglcs with specific β-glucosidases after tissue damage^22^,^23^. HCN is an acute respiratory toxin to almost all eukaryotic organisms^24^; however, its odour or taste alone does not repel all insect enemies, e.g. not predatory ants^25^. Thus, the absence of a dual toxic/repellent role of HCN may have spurred evolution of additional physical and chemical defences in CNglc-containing secretions. This is exemplified by defence droplets in closely related *Z. trifolii* larvae, which possess strong repellent properties against various vertebrate and invertebrate predators^16^,^26^. This observation raises questions about the molecular and chemical basis of repellence and deterrence, and whether other substances and mechanisms apart from CNglcs in the defence droplets are required for their effectiveness.

In this study we used a multidisciplinary approach to advance our general knowledge on both physical and chemical defence mechanisms in insect secretions using *Z. filipendulae* larvae as the experimental system. The effectiveness of their defence droplets was tested on two potential predators and most of the constituents in the defence droplets and their mode of action were identified and analysed. Our study shows that the mode of action of the defence droplets depends on the type of predator as well as on the degree of irritation or injury the larvae are subjected to.

## Results

### Wetting capacity and viscosity of the defence droplets

**Figure 2:**
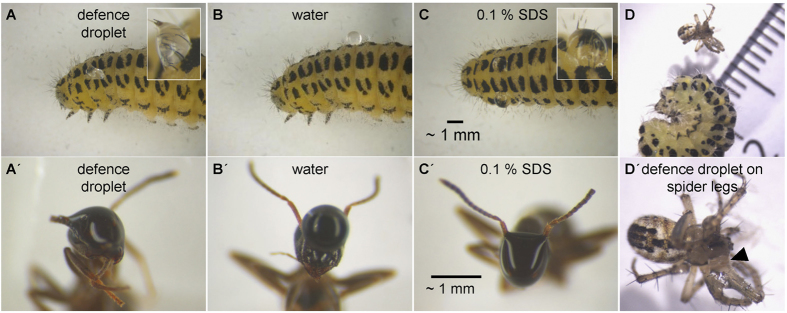
Defence droplets glue together mouthparts, legs and sensory organs of potential predatory arthropods. (**A**) After physical irritation, *Z. filipendulae* larvae release viscous defence droplets which wet the hydrophobic cuticle of ants such as *M. rubra* (**A′**) including their mouthparts such as mandibles and sensory organs such as antennae. (**B + B′**) Water does not wet the hydrophobic cuticle neither of *Z. filipendulae* nor of the ant. (**C + C′**) A 0.1% SDS solution wets the cuticle of *Z. filipendulae* larvae as well as of ants. (**D**) Spiders such as *M. acalypha* may also induce the release of defence droplets, (**D′**) which glue their legs and immobilise them (arrow).

Defence droplets were able to wet the *Z. filipendulae* cuticle, in contrast to water (Fig. 2A,B). Similarly, a 0.1% solution of the surfactant SDS (sodium dodecyl sulfate) was able to wet the *Z. filipendulae* larval cuticle (Fig. 2C). The effect of defence droplets on potential predators was illustrated using the common red ant *Myrmica rubra* (Hymenoptera: Formicidae) and the cricket-bat orb weaver spider *Mangora acalypha* (Araneae: Araneidae). Defence droplets completely wet the hydrophobic cuticle of the ant head including mouthparts such as mandibles and sensory organs such as antennae (Fig. 2A′). Around five minutes after the wetting, the defence droplets became increasingly sticky and glued the ants and their appendages together and immobilized them. Similarly, the surfactant 0.1% SDS wet the ant head including the mandibles (Fig. 2C′). In contrast, water was not able to wet the head cuticle of the ant (Fig. 2B′). In experiments with *M. acalypha* (Fig. 2D) the defence droplets were observed to effectively glue together the legs of the spider which resulted in complete immobilisation and finally death (Fig. 2D′).

### Proteins and peptides in the defence droplets

**Figure 3:**
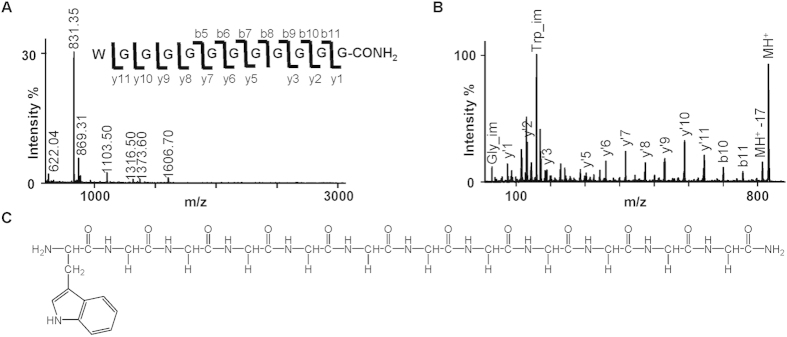
Mass spectrometric *de novo* sequencing of a major peptide from defence droplets. (**A**) MALDI-TOF-MS spectrum of defence droplets directly deposited on target. (**B**) MS/MS fragment spectrum of the MH^+^ 831.35 ion from the MS spectrum. Only annotated y- and major b-ions as well as immonium (im) ions are labelled in the spectrum for clarity. The sequence is shown with annotated fragment ions below the spectrum according to nomenclature of 61. The C-terminal Gly is α-amidated. (**C**) Primary structure of the peptide.

Since viscous secretions from the few lepidopteran species investigated are proteinaceous^26^,^27^, the protein composition of the *Z. filipendulae* defence droplets was determined. To tentatively characterize the proteins present in the defence droplets, LC-MS/MS (Liquid chromatography-tandem mass spectrometry) based amino acid sequencing and subsequent database searches were carried out. A total of 66 different proteins were identified (Table 1). Protease inhibitors, proteases and oxidases were the most abundant types of proteins in the defence droplets constituting 32 of the 66 identified proteins. The presence of peptides in the defence droplets was investigated using MALDI-TOF-MS/MS (Matrix assisted laser desorption/ionization time-of-flight tandem mass spectrometry) in the mass region 600–5000 Da. This approach revealed a single abundant peptide with a mass of 831.35 (MH^+^) (Fig. 3A), which based on its fragmentation spectrum (Fig. 3B) was identified as the peptide H-WG_11_-NH_2_ (i.e. tryptophan followed by an 11-omer of glycine) with a C-terminal α-amidation (Fig. 3C). The H-WG_11_-NH_2_ peptide was not detected in the larval haemolymph. A transcript corresponding to this sequence could not be found in any of the three available *Z. filipendulae* transcriptomes^21^,^28^. This indicates that the peptide in *Z. filipendulae* originates via post translational processing. To find a possible match for the peptide in other organisms, the sequence was searched against UniProtKB using Peptide Match^29^ and against the NCBI non-redundant protein sequences database using the BLASTP algorithm. This approach resulted in sequence hits from several different organisms with 100% coverage and 100% sequence identity. However, all hits were part of larger protein sequences such as the predicted secreted protein XP_002406990 from the black-legged deer tick *Ixodes scapularis* (Ixodida: Ixodidae), corroborating that the H-WG_11_-NH_2_ peptide may originate from post translational processing.

### Metabolite profiling of the defence droplets and haemolymph

To identify non-proteinaceous and small metabolites, an unbiased analysis of the metabolite composition of the defence droplets and haemolymph was carried out using GC-MS (Gas chromatography-mass spectrometry). An earlier comparison of the metabolite profile of the defence droplets and the haemolymph had shown that both defence droplets and haemolymph contain high amounts of the CNglcs linamarin and lotaustralin^17^. However, these CNglcs can only be detected by LC-MS and not by GC-MS due to their inherent thermal instability. In both defence droplets and haemolymph, the main metabolite components detected were carbohydrates and free amino acids (Table S1). Organic acids and sterols were present in both tissues in minor amounts, while a few higher alkanes and terpenes were detected in the haemolymph. Eighteen different carbohydrates were identified in the defence droplets including mono- and di-saccharides and sugar alcohols (Table S1). Glucose constituted the vast majority of all small metabolites in the defence droplets with a 93.5% relative abundance. The second most abundant carbohydrate was myo-inositol with a relative abundance of almost 1%. In the haemolymph, 15 different carbohydrates were detected with the major constituents being allose (22%) and trehalose (18.7%). Four amino acids were detected in the defence droplets of which the non-protein amino acid β-cyanoalanine was by far the most abundant one (1.0% relative abundance). Similar β-cyanoalanine content (0.9%) was found in the haemolymph, but the diversity of amino acids was higher in this tissue (Table S1).

### Testing β-glucosidase activity and HCN release from the defence droplets and haemolymph

**Figure 4:**
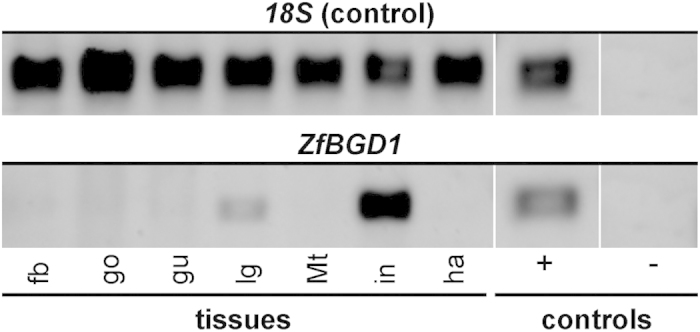
The β-glucosidase *ZfBGD1* is specifically expressed in the integument. Semi-quantitative RT-PCR on *ZfBGD1* using cDNA derived from different larval tissues of *Z. filipendulae*. Abbreviations: fb-fat body, go-gonads, gu-gut, lg-labial glands, Mt-Malpighian tubules, in-integument, ha-haemolymph. As positive controls 18S rRNA primers and whole larval cDNA (+) were used; water (−) as template constituted the negative control. Note that the integument contains cuticular cavities, which harbour the defence droplets.

Since *Z. filipendulae* larvae emit HCN^30^,^31^, it was anticipated that the specific β-glucosidases required to hydrolyse linamarin and lotaustralin would be located in the released droplets. The only β-glucosidase detected in the defence droplets was ZfBGD1 (Table 1). A partial nucleotide sequence from *ZfBGD1* was found in the *Z. filipendulae* larval transcriptome and a full-length sequence was obtained by RACE (Rapid amplification of cDNA ends)-PCR. Semi-quantitative RT (Reverse transcriptase)-PCR showed that *ZfBGD1* was predominantly expressed in the larval integument, which contains the cuticular cavities harbouring the defence droplets (Fig. 4). To elucidate whether the ZfBGD1 enzyme was involved in CNglc-hydrolysis, it was expressed in *Sf9* insect cells and purified. Recombinant ZfBGD1 was demonstrated to be functionally active by its ability to hydrolyse the generic β-glucosidase substrate 4-methylumbelliferyl β-D-glucopyranoside (MUglc) at a rate of 17.8 nmol MU/h/μg ± 1.7 s.e.m. Surprisingly, ZfBGD1 did not show any hydrolytic activity towards linamarin and lotaustralin (0.0 nmol HCN/h/μg). In agreement with this result, the defence droplets exhibited β-glucosidase activity against MUglc (10.1 nmol MU/h/μl), but none against linamarin nor lotaustralin (0.0 nmol HCN/h/μl). This finding was supported by Raman spectroscopy, which demonstrated that the CNglcs in the droplets stayed intact over time as monitored by the diagnostic and well-resolved absorption band near 2245 cm^−1 ^^32^ corresponding to the CN bond stretching of the nitrile group in the CNglcs (Fig. S1). Incubation of the defence droplets with an enzyme preparation purified from *Z. filipendulae* haemolymph devoid of CNglcs resulted in the release of 57.3 ± 7.0 nmol HCN/h/μl. This is significantly higher than the HCN release obtained from the same amount of crude haemolymph (30.8 ± 8.4 nmol/h/μl; *P* = 0.035, Student's one-tailed t-test).

### Formation of crystalline-like precipitates in defence droplets

**Figure 5:**
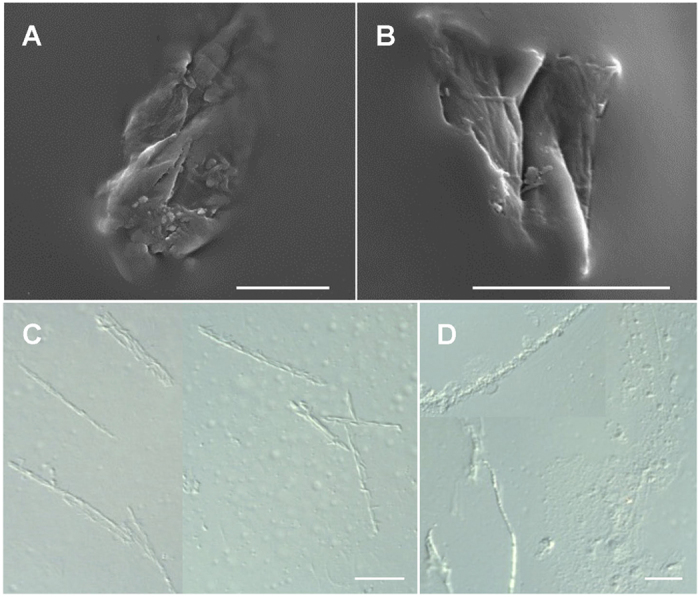
Formation of crystalline-like precipitates in defence droplets after their release. Precipitates composed of particles with dimensions as large as 50 × 30 μm and irregular shapes ranging from almost oval (**A**) and triangular with sharp edges (**B**), as seen by electron microscopy, to crystal-like needle shapes (**C,D**) that are up to 50 μm long as seen via differential interference contrast microscopy. The scale bar equals 20 μm.

Around 5 minutes after release, the defence droplets began to harden, i.e. they changed from being highly viscous to sticky to finally solid. At this point the *Z. filipendulae* larvae were no longer able to reabsorb the droplets. The hardening process was completed within a circa 10 min period. The hardened structures were analysed in more detail via scanning electron and differential interference contrast microscopy. This revealed that the hardening of defence droplets resulted in the formation of largely insoluble crystalline-like precipitates (Fig. 5). The dimensions of the largest crystalline precipitates reached 50 × 30 μm with irregular shapes ranging from almost oval (Fig. 5A) and triangular with sharp edges (Fig. 5B) to needle-like crystals that were up to 50 μm long (Fig. 5C,D).

### Water evaporation and build-up of antiparallel β-sheets during droplet hardening

**Figure 6:**
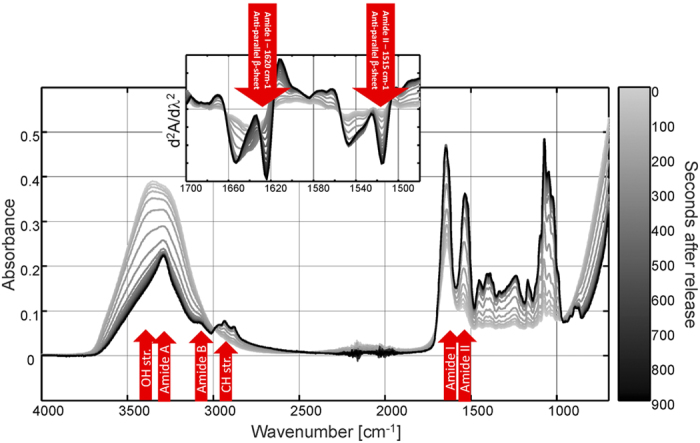
FT-IR spectroscopy indicates spectral changes during hardening of the defence droplets. The insert shows the second derivative spectra of the amide I and II region. The second derivative spectrum has the advantage of an increased apparent resolution which can reveal the secondary structure elements of the proteins. Note that when the absorbance peaks go up in the normal spectrum they go down in the second derivative spectrum^62^. At time zero the droplets contain water, peptides and sugars (1000–1200 cm^−1^). The grey scale indicates how soon after droplet release the sample was run. The kinetic profile shows that water is evaporated and that the proteins become concentrated and highly ordered in antiparallel β-sheets.

The possible involvement of proteins and the importance of their secondary structures during hardening and precipitate formation in defence droplets were investigated using Fourier-transform infrared spectroscopy (FT-IR). The measurements documented that the amide I and amide II bands at 1622 cm^−1^ and 1515 cm^−1^, respectively, increased significantly around 5 minutes after defence droplet release when the hardening process started, and reached a maximum after 5 more minutes when the hardening process was completed (Fig. 6). Fresh defence droplets that were precipitated in 100% methanol showed similar characteristic amide I and II FT-IR bands at 1622 cm^−1^ or 1521 cm^−1^, respectively (Fig. S2). The increase of amide I and II band intensities at 1622 cm^−1^ and 1515 cm^−1^ indicates a strong build-up of antiparallel β-sheet structures^33^. The simultaneous decrease of the OH-stretching band at 3330 cm^−1^ indicates loss of water concomitant to the hardening process of the defence droplets (Fig. 6). To determine whether these changes were directly related to the presence of the H-WG_11_-NH_2_ peptide, the peptide was obtained by chemical synthesis. FT-IR measurements using the synthesised peptide solubilized in distilled water did not result in build-up of antiparallel β-sheet structures. Instead, the peptide exhibited amide I and amide II bands at 1641 cm^−1^ and 1555 cm^−1^, respectively, which indicates a rather unordered structure^33^. Accordingly, the *in vitro* experiments do not indicate that the structural changes observed in defence droplets are directly linked to the presence of H-WG_11_-NH_2_. However, it is conceivable that other molecules or ions present in the droplets affect the structure of the peptide *in vivo*, possibly leading to formation of the antiparallel β-sheets.

## Discussion

The chemical composition of defence secretions in arthropods and insects may be complex^7^,^8^ with the constituents packed into a viscous substance as reported for some species of centipedes and termites^13^,^34^. In line with these previous studies, our studies on *Z. filipendulae* demonstrate versatile defence functions of droplets produced by a Lepidopteran larva. The first physical barrier a predator of *Z. filipendulae* larvae encounters is the high viscosity of the defence droplets, which serve to wet and glue together the hydrophobic cuticles such as of mandibles, legs and sensory organs as shown here for an ant and a spider (Fig. 2). Droplets of *Z. filipendulae* have surfactant-like properties, i.e. their surface tension is reduced in comparison to water. Similarly, the surfactant-like properties of oral secretions from the lepidopteran larva of *Spodoptera exigua* are able to wet the hydrophobic cuticle and to glue the antennae of ants, efficiently preventing further attacks^27^. The ability of secretions to wet locomotory and sensory structures is highly effective as a defence system, since it may immobilise predators as well as impair proper sensory functioning^27^. Viscous secretions may also serve to glue and block other susceptible structures like insect sensillae and spiracles needed for respiration^13^.

Viscosity and stickiness of lepidopteran defensive secretions may be caused by the presence of proteins^26^,^27^. Proteomic profiling of the *Z. filipendulae* defence droplets confirmed a highly proteinaceous content, which likely contributes to the viscosity and stickiness of the defence droplets. The glycine-rich peptide H-WG_11_-NH_2_ in particular was highly abundant in the defence droplets (Fig. 3), but was absent from the non-viscous haemolymph. H-WG_11_-NH_2_ may be an important functional component of the defence droplets because the amino acid glycine is characterised by intermediate hydrophobicity and thus able to form intermolecular interactions with water and other constituents in the droplets thereby contributing to protein solubility^35^.

Given that free glucose constitutes almost 94% of all small metabolites detected in the defence droplets (Table S1), glucose may also contribute to the viscosity/stickiness of defence droplets in addition to the proteinaceous content. The polysaccharide dextran and its monomer glucose are known to decrease the surface tension of solutions^36^, which may explain why *Z. filipendulae* larvae are able to wet and subsequently glue together the hydrophobic structures of predatory arthropods (Fig. 2). In contrast, in the non-viscous haemolymph, other sugars such as allose and trehalose were dominant, whereas glucose constituted a minor component. Trehalose, the non-reducing disaccharide of glucose, is often the main sugar in the haemolymph of insects^37^, whereas the monosaccharide allose is a rare, possibly so far undetected component in insect haemolymph^38^,^39^.

The largest groups of the proteins identified in the defence droplets were protease inhibitors, proteases and oxidases (prophenol oxidases, peroxidases) (Table 1). Because of their defensive properties, these proteins have been reported as key components employed by insects and plants to fend off diverse enemy species^40^,^41^,^42^. Defence secretions of grasshoppers may contain a diversity of protease inhibitors directed against entomopathogenic fungi^43^. Ingestion of plant tissue containing proteases may severely disrupt the peritrophic membrane in the insect midgut resulting in impaired nutrient utilization and thus reducing growth by 60–80%^44^. Glandular defence secretions in some *Chrysomelina* leaf beetles may contain oxidases, which catalyse the generation of salicin-based feeding deterrents^45^. In a similar fashion, predators of *Z. filipendulae* may suffer damage from the defence proteins present in the defence droplets, if ingested.

β-Cyanoalanine was found in both the defence droplets and the haemolymph (Table S1); in the droplets it was the most abundant amino acid and the third most abundant small metabolite. β-Cyanoalanine is a detoxification product of HCN, catalysed by β-cyanoalanine synthases, and occurs in many lepidopteran species^46^,^47^. However, for vertebrate species β-cyanoalanine can also function as a neurotoxin^48^. Rats and chickens show signs of intoxication such as hyperirritability, tremors, convulsions and ultimately death only 13 h (rats) or 4 d (chickens) after being fed a diet containing 1.5% or 0.25% β-cyanoalanine, respectively^49^. Thus, apart from the toxicity residing in linamarin and lotaustralin, the β-cyanoalanine in the defence droplets of *Zygaena* may also be toxic.

The high content of CNglcs in the defence droplets^17^ and the finding that *Z. filipendulae* larvae release HCN^30^,^31^ intuitively suggested that a specific β-glucosidase should also be present in the defence droplets, perhaps only activated upon droplet release. The proteomic analysis revealed ZfBGD1 as the only β-glucosidase in the defence droplets (Table 1). ZfBGD1 expressed in insect cells catalysed hydrolysis of the generic β-glucosidase substrate MUglc, whereas it did not catalyse hydrolysis of the glucosidic bond in linamarin or lotaustralin. The same result was obtained when defence droplets were incubated with these substrates. Thus, the lack of HCN release from the defence droplets matches the inability of ZfBGD1 to hydrolyse CNglcs. Defence secretions that release HCN have been found in other Lepidopteran species such as thyridid caterpillars (*Calindoea trifascialis*)^50^, in Coleopteran species (*Paropsis atomaria*)^51^ and in species from other arthropod orders such as Chilopoda (*Geophilus vittatus*)^34^ and Diplopoda (e.g. *Polydesmus complanatus*)^52^. However, no specific β-glucosidase involved in CNglc catabolism has been characterised in these species yet.

Raman spectroscopy carried out on the defence droplets showed that the signal specific to the nitrile group of the CNglcs remained intact over time (Fig. S1). This provided independent support to the finding that the CNglcs present in the defence droplets were stable and not hydrolysed by ZfBGD1. The release of defence droplets by *Z. trifolii* has been shown to repel vertebrate predators such as starlings and lizards^16^. If the absence of a β-glucosidase that can hydrolyse CNglcs is a general phenomenon in the defence droplets of *Zygaena* species, the intact CNglcs may instead function as deterrents based on their bitter taste^46^,^53^. Secretion of bitter chemicals minimize injuries and predation because they enable predators to assess palatability based on taste and to reject prey due to bitterness aversion^53^,^54^. In the rare case that *Zygaena* larvae are swallowed by predators, the CNglcs in the defence droplets may be hydrolysed into toxic HCN in the predator’s gut by digestive β-glucosidases, which are at least partly active towards linamarin or lotaustralin. Rats given either a single oral dose of linamarin (500 mg/kg) or a lower oral dose of cyanide (6 mg/kg) showed signs of intoxication such as cardiac arrhythmias and respiratory changes indicating that the ingested linamarin was at least partly hydrolysed during passage through the rat’s gut^55^.

We have demonstrated that the defence droplets of *Z. filipendulae* do not release HCN *per se*. Therefore, the moderate amount of HCN released from *Z. filipendulae* larvae as reported in other studies^30^,^31^ may be generated in the haemolymph, which also contains high amounts of linamarin and lotaustralin (~11 μg per μl)^17^. In our study, *Z. filipendulae* haemolymph was found to release HCN as expected from a β-glucosidase catalysed reaction. Interestingly, an enzyme preparation from haemolymph devoid of CNglcs and spiked with defence droplets released double amounts of HCN (~60 nmol/h/μl) than the same volume of crude haemolymph (~30 nmol/h/μl). These values are up to several hundred fold higher than HCN release from whole, alive and non-irritated *Z. filipendulae* larvae as found in^30^,^31^. These findings rule out the presence of a β-glucosidase inhibitor in the defence droplets and indicate that only a minor fraction of HCN produced in the larval haemolymph is released to the environment. Moreover, since many predators are expected to injure the larva, exuding haemolymph from a wound will most likely mix with defence droplets onto the cuticle resulting in the release of toxic and deterring HCN. Consequently, high amounts of HCN are produced: (i) outside of the larval body and (ii) only on demand, i.e. after attack and tissue damage and (iii), after mixing of haemolymph-located enzymes and droplet-based substrates.

When wetted by viscous secretions, the appendages of arthropod predators may become stuck together. To avoid this, some predators perform cleansing behaviour^27^. If the appendages remain stuck together the predator may die, especially when the secretion hardens after exposure to air as reported in this study and as previously observed with secretions from termites and centipedes^13^,^34^. The hardening process in the *Z. filipendulae* defence droplets starts approximately 5 minutes after droplet release, is completed after 5 more minutes and is accompanied by the formation of crystalline-like precipitates (Fig. 5). The hardened secretions may also act as physical abrasives and digestion inhibitors to chewing insect predators because their sharp edges and needle-like shapes are likely to wear and tear mandibles and thereby interfere with digestion. Similarly, calcium oxalate crystals in many plant species have pronounced abrasive effects on mandibles of chewing caterpillars and ingestion of these crystals leads to reduced growth and increased mortality^56^.

Antiparallel β-sheets are abundant secondary structures in globular proteins and often involved in protein aggregation^57^. Formation of antiparallel β-sheets was observed in the defence droplets starting and finishing at the same time as the hardening process (Fig. 6). Enhanced protein concentrations as a result of water evaporation probably stimulated aggregation and final hardening of the defence droplets. In the silkworm *Bombyx mori* (Lepidoptera), the viscosity of the silk solution is increased by increasing the concentration of protein in the silk gland^35^. Similarly the viscous defence secretions of the African termite *Odontotermes badius* harden due to water evaporation, which may be accompanied by denaturation of endogenous proteins^58^. The finding that the amide I and II regions of the FT-IR spectra of defence droplets denatured with methanol were identical and similar, respectively, to the spectra obtained from the final state of hardened defence droplets (Fig. S2) strongly support that the hardening of the *Z. filipendulae* defence droplets is the combined result of protein aggregation, caused by formation of antiparallel β-sheets, and water evaporation.

This study illustrates that the defence droplets of *Z. filipendulae* larvae harbour a battery of versatile and composite physical and chemical defence systems to be deployed against predators, and provides insights into the underlying mechanisms of the defence. The defence droplets situated in cuticular cavities of the larvae can be regarded as morphological depositories, physically separated from the rest of the body. They enable the larvae to accumulate diverse defence components without the risk of self-intoxication, and at the same time, predators are immediately exposed to this defence weapon after attack.

## Methods

### Predator assays

Larvae of *Z. filipendulae* were collected from a natural population in the south-west of Taastrup (55.65°N, 12.30°E), Denmark. As examples of potential *Z. filipendulae* larvae predators, individuals of the common red ant *M. rubra* (Hymenoptera: Formicidae; six biological replicates) and the cricket-bat orb weaver spider *M. acalypha* (Araneae: Araneidae; two biological replicates) were collected from natural populations in Frederiksberg (55.68°N, 12.51°E) and Taastrup, Denmark. 0.5 μl of defence droplets from fifth instar *Z. filipendulae* larvae (out of seven instar stages), distilled water or 0.1% SDS was applied on the head and mandibles of the ants or on the cuticles of *Z. filipendulae* fifth larval instar. 2 μl defence droplets were applied to the legs of the spiders. The effects of the defence droplets were recorded using a digital camera (Nikon DS-Fi1) mounted on a light microscope (Leica Wild M3Z).

### LC-MS/MS

To identify proteins present in the defence droplets, 1 μl fresh defence droplet from fifth instar *Z. filipendulae* larvae was digested with trypsin after denaturation, reduction and alkylation of Cys. An aliquot of the resulting proteolytic digest was desalted and subjected to nano-reverse-phase LC-MS/MS. Two biological replicates were analysed. The amino acid sequence data obtained were searched against a *Z. filipendulae* transcriptome database^28^ translated in six reading frames to identify protein sequences. The protein sequences were functionally annotated by BLAST searches (Protein BLAST and BLASTX against all non-redundant GenBank CDS, and CD BLAST). For further details see supplementary methods.

### MALDI-TOF-MS/MS

1 μl fresh defence droplet from the fifth instar of a *Z. filipendulae* larva was mixed with 100 μl water. Immediately after dilution, 1 μl of the resulting solution was deposited on a MALDI target with matrix solution and subjected to MALDI-TOF-MS and MS/MS. Four biological replicates were analysed. For further details see supplementary methods.

### GC-MS

For sample preparation, 10 μl defence droplets or 10 μl haemolymph were collected from punctured prolegs. For both tissues, three biological replicates from the same fifth instar of a *Z. filipendulae* larva were analysed. Each sample replicate was mixed with 90 μl 100% methanol. After centrifugation at 21.000 g for 3 min, 80 μl of supernatant was dried and derivatized by addition of 40 μl trimethylsilyl cyanide^59^. Splitless mode was used for sample injection into GC-MS. GC-MS data acquisition parameters were according to Khakimov *et al*.^59^ with few modifications: GC oven equilibration time was 3 min, end temperature was 310 °C and hold time 8 min. After 6.6 min of solvent delay, mass spectra were recorded within 50–500 *m*/*z* range at the scanning frequency of 3.2 scans sec^−1^. All metabolites were tentatively identified at level 2 according to the Metabolomics Standards Initiatives using EI-MS library match of >800; except for β-cyanoalanine (Sigma-Aldrich C9650) which was identified using an authentic standard (1 μl 75 μM). Relative areas of peaks were calculated using ChemStation software (version: E.02.02.1431, Agilent Technologies, Inc.) and relative percentages (RP), and relative standard deviations (RSD) of peaks were calculated from replicate samples of both tissue types.

### Molecular cloning, heterologous expression and RT-PCR

RNA was extracted from the fifth instar of a *Z. filipendulae* larva using the RNeasy^®^ Mini Kit (Qiagen). cDNA was generated using iScript™ cDNA Synthesis Kit (Biorad). To obtain the full-length open reading frame, 5′-RACE was carried out using SMARTer^®^ RACE cDNA Amplification Kit (Clontech) and the gene specific primer ZfBGD1GSP and ZfBGD1NGSP. The product was purified using QIAquick^®^ PCR purification kit (Qiagen) and sequenced (Eurofins Genomics). The native open reading frame of *ZfBGD1* was Phusion^®^ (Finnzymes) polymerase-amplified using the primers ZfBGD1orfF and ZfBGD1orfR, cloned into pCR-Blunt II-TOPO^®^ (Life Technologies) and sequenced (ENA accession number: LN851681). *ZfBGD1* was amplified using primers with restriction sites and a C-terminal HIS-tag (ZfBGD1Fxma, ZfBGD1RHISnot), purified, and subcloned into pAcGP67A (BD Pharmingen) containing a secretion signal. After transformation into chemically competent One Shot^®^ TOP10 *E. coli* (Life Technologies), the plasmid was purified using QIAprep^®^ Spin Miniprep Kit (Qiagen) and sequenced. The pAcGP67A-*ZfBGD1*-HIS construct was mixed with Baculo-Gold DNA™ (BD Pharmingen) and co-transfected with *Sf*9 insect cells (Life Technologies). For details of heterologous expression, dialysis of the culture medium containing ZfBGD1 and Ni-NTA purification see^60^. For RT-PCR, fifth instar *Z. filipendulae* larvae were dissected into integument (i.e. cuticle with epidermal cell layer) (5), haemolymph (5), gut (5), fat body (1), gonads (1), labial glands (1) and Malpighian tubules. The number of biological replicates is indicated in brackets. RNA purification and cDNA synthesis were carried out as described. 25 cycles were run on each tissue-specific cDNA using the primers ZfBGD1seq3f and ZfBGD1orfR. As positive controls, 18S rRNA primers and whole larval cDNA were used; water as template constituted the negative control. Each amplicon was separated by agarose gel electrophoresis and visualised. For all primers used, see Table S2.

### β-Glucosidase assays

Purified extracts of ZfBGD1 were measured for protein concentration on a NanoDrop ND-1000 (Thermo Scientific) and assayed for β-glucosidase activity using the generic substrate 4-methylumbelliferyl β-D-glucopyranoside (MUglc, Sigma M3633) and the CNglcs linamarin (Sigma-Aldrich 68264) or lotaustralin. 1 μg of ZfBGD1was incubated with 500 μM of each substrate in 240 μl 60 mM citric acid buffer (pH 6) for 60 min at 37 °C. Release of hydrolysis products, i.e. aglucones, was measured on a microplate reader SpectraMax M5 (Molecular Devices) and quantified based on a corresponding standard curve. For hydrolysis of MUglc, the reaction was stopped by adding one volume ice-cold 100% methanol. The fluorogenic aglucone 4-methylumbelliferone (MU) was measured by reading fluorescence with excitation at 366 nm and emission at 454 nm, and compared to standards of MU diluted in 50% methanol. For hydrolysis of CNglcs, HCN release was measured as described elsewhere^31^. As positive control, haemolymph without CNglcs, but containing specific β-glucosidase activity, was used. Therefore, haemolymph was collected from punctured prolegs of ten *Z. filipendulae* larvae in the fifth instar. A PD-10 desalting column with Sephadex^®^ G-25 medium (GE Healthcare) was used to purify crude haemolymph from CNglcs. Desalted haemolymph was applied to a Vivaspin^®^ 6 (GE Healthcare) sample concentrator with a cut-off at 30 kDa. Concentrated haemolymph was diluted to the initial volume with ice-cold 60 mM citric acid buffer (pH 6). The physiological pH of haemolymph 6.3 ± 0.1 s.d. (ten biological replicates) was determined on a Hanna 211 pH meter with the pH electrode HI 1131B (Hanna Instruments).

### Release of HCN

For analysis of general β-glucosidase activity in the defence droplets derived from fifth instar larvae, 1 μl droplet was incubated at 30 °C for 1 h in 240 μl 60 mM citric acid buffer (pH 6) containing 200 μM MUglc. Release of the aglucone MU was quantified as described above. To determine whether the β-glucosidase from the defence droplets could hydrolyse the CNglcs present in the defence droplets *in vivo*, 1 μl defence droplet was either incubated alone or together with 1 μl enzyme preparation purified from larval haemolymph of the same instar and devoid of CNglcs as positive control at 30 °C for 1 h in 200 μl 60 mM citric acid buffer (pH 6). For comparison to HCN emission from the haemolymph, 1 μl crude haemolymph was incubated with 1 μl haemolymph devoid of CNglcs at the same conditions. In each case, three biological replicates were analysed and HCN release was measured as described elsewhere^31^. Mean values with standard error were calculated and tested for significance using Student’s t-test (one-tailed, SigmaPlot 12).

### Microscopy

1 μl defence droplet freshly taken from a fifth instar of *Z. filipendulae* larva was air-dried on a microscope glass slide, placed on a carbon conductive adhesive tab mounted to an aluminium stub and sputter coated with a 1:1 gold-palladium mixture. Micrographs of the sample were taken using scanning electron microscopy at 10–15 kV on a Quanta 200 SEM (FEI Company). To analyse the inner structures of untreated and thus uncoated defence droplets, differential interference contrast microscopy was applied. Therefore, 1 μl fresh defence droplet was placed on a glass slide with immersion and then covered by a cover slip. After hardening and formation of crystalline-like structures, pictures were taken on a Leica DM 5000B fluorescence microscope using differential interference contrast. For each microscopy technique, two biological replicates were analysed.

### Spectroscopy

Fourier-transform infrared spectra (FT-IR) were collected on an Arid-Zone MB100 FT-IR instrument (ABB Bomen, Quebec, Canada) using an attenuated total reflectance device with a triple-bounce diamond crystal. The IR spectra were recorded in the range from 4400–750 cm^−1^ using a spectral resolution of four cm^−1^. Droplets were freshly taken from fifth instar *Z. filipendulae* larvae. Test samples were either 2 μl of *i*.) defence droplets (three biological replicates), *ii*.) 250 mM H-WG_11_-NH_2_ (purity >90%, synthesized by JPT Peptide Technologies) diluted in distilled water (three technical replicates) or *iii*.) defence droplets diluted 1:10 in 100 % methanol from which the pellet, i.e. precipitate, was taken (three biological replicates). Spectra were recorded every 30 s with eight accumulations over a period of 15 min. Each spectrum represented the average of 32 scans compared to the background (64 scans) collected with the empty crystal and stored as absorbance spectra. CNglcs can be specifically measured by Raman spectroscopy due to the nitrile group, which has a well-resolved CN stretching band near 2245 cm^−1^ 32. Raman spectra were collected on a RamanRxn1 instrument equipped with an Invictus™ 785 nm near-infrared diode laser (Kaiser Optical Systems Inc., MI, USA). Data were collected using a single holographic grating and a thermoelectric cooled charge-coupled device detector, operated at −40 °C. The probe was an Mk II filtered probe attached to a Leica microscope (Leica microsystems) equipped with a 10× objective. The Raman spectra of 2 μl defence droplets (three biological replicates) were recorded every 30 s with eight accumulations of 1 s exposure for a total period of 15 min. The spectra were stored as Raman shifts in the range 3500–0 cm^–1^.

## Additional Information

**Accession code**: ZfBGD1 (ENA accession number: LN851681).

**How to cite this article**: Pentzold, S. *et al*. Lepidopteran defence droplets - a composite physical and chemical weapon against potential predators. *Sci. Rep*. **6**, 22407; doi: 10.1038/srep22407 (2016).

## Supplementary Material

Supplementary Information

## Figures and Tables

**Figure 1:**
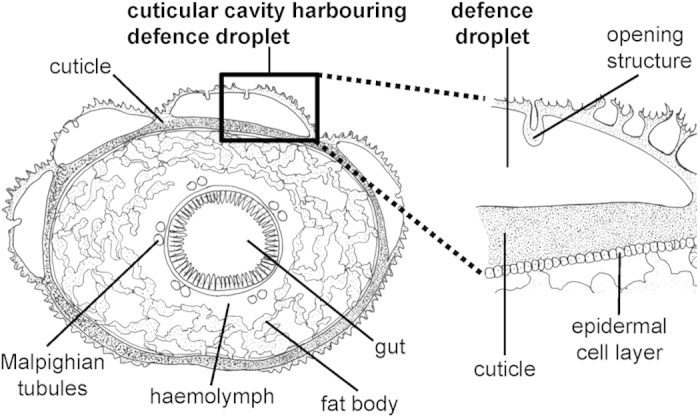
Cross section of *Zygaena* larvae showing segmentally arranged cuticular cavities harbouring defence droplets. There are up to eight of these relatively large cavities per segment, except the first and the two last segments. Roughly in the centre of the cavity the cuticle is folded and reaches into the interior of the cavity to form a thin opening structure. After mild physical irritation, defence droplets are released. For further morphological details see^16^. The Figure is adapted from^21^.

**Table 1:** Proteins identified as present in the defence droplets of *Z. filipendulae*.

***Protease inhibitors (18):***	Hormone/odorant binding protein (5)
Serine protease inhibitor (17)	C-type lectin (3)
Trypsin inhibitor (1)	Aldo-keto reductase (2)
***Proteases (10):***	Peptidyl prolyl cis-trans isomerase (2)
Serine protease (7)	Phosphatidylethanolamine binding (1)
Metalloprotease (2)	Transferrin (1)
Cysteine protease (1)	Apolipoprotein D (1)
***Oxidases (4):***	Calcyphosine-like (1)
Prophenoloxidase (2)	Cuticular protein tweedle motif (1)
Peroxidase (1)	Split ends-like (1)
Sulfhydryl oxidase (1)	Calcyphosine-like (1)
***Hydrolases (8):***	γ-interferon lysosomal thiol reductase (1)
Phosphatase (2)	Yellow-C (1)
Ribonuclease (1)	Yellow-D (1)
Lipase (1)	Ommochrome-binding protein-like (1)
β-glucosidase ZfBGD1 (1)	Plexin domain-containing protein (1)
α-amylase (1)	Chemosensory protein (1)
Chitinase-like (1)	Unknown (1)
α-trehalase (1)	

In total, 66 different proteins were identified by LC-MS/MS. Defensive proteins such as protease inhibitors, proteases and oxidases were most abundant. MS and MS/MS tolerances were set to 10 ppm and 0.8 Da, respectively; false discovery rate set to 1%. The annotated functions are according to BLAST searches. Number in brackets indicates number of different proteins identified. For further details see Table S3 and supplementary methods.
