# Supplementary material for: Lepidopteran defence droplets - a composite physical and chemical weapon against potential predators
Source: Sci Rep. 2016 Mar 4;6:22407. doi: 10.1038/srep22407 (PMC4778013; doi:10.1038/srep22407)
Supplement: Supplementary Information [file srep22407-s1.docx]

**Supplementary information**

**Lepidopteran defence droplets - a composite physical and chemical weapon against potential predators**

Stefan Pentzold^1§*^, Mika Zagrobelny^1^, Bekzod Khakimov^2^, Søren Balling Engelsen^2^, Henrik Clausen^3^, Bent Larsen Petersen^4^, Jonas Borch^5^, Birger Lindberg Møller^1,6^, Søren Bak^1^

*^1^Plant Biochemistry Laboratory and Villum Research Center ‘Plant Plasticity’, Copenhagen Plant Science Center, Department of Plant and Environmental Sciences, ^2^Spectroscopy and Chemometrics, Department of Food Science, ^3^Copenhagen Center for Glycomics, Department of Cellular and Molecular Medicine, ^4^Plant Glycobiology, Department of Plant and Environmental Sciences, University of Copenhagen, Copenhagen, Denmark; ^5^Department of Biochemistry and Molecular Biology, University of Southern Denmark, Odense, Denmark; ^6^Carlsberg Laboratory, Copenhagen, Denmark;*

*^§^Present address: Max Planck Institute for Chemical Ecology, Jena, Germany*

*Corresponding author.

Email: [spentzold@ice.mpg.de](mailto:spentzold@ice.mpg.de)

Phone: ++49(0) 3641-571267

**Supplementary figures**

**
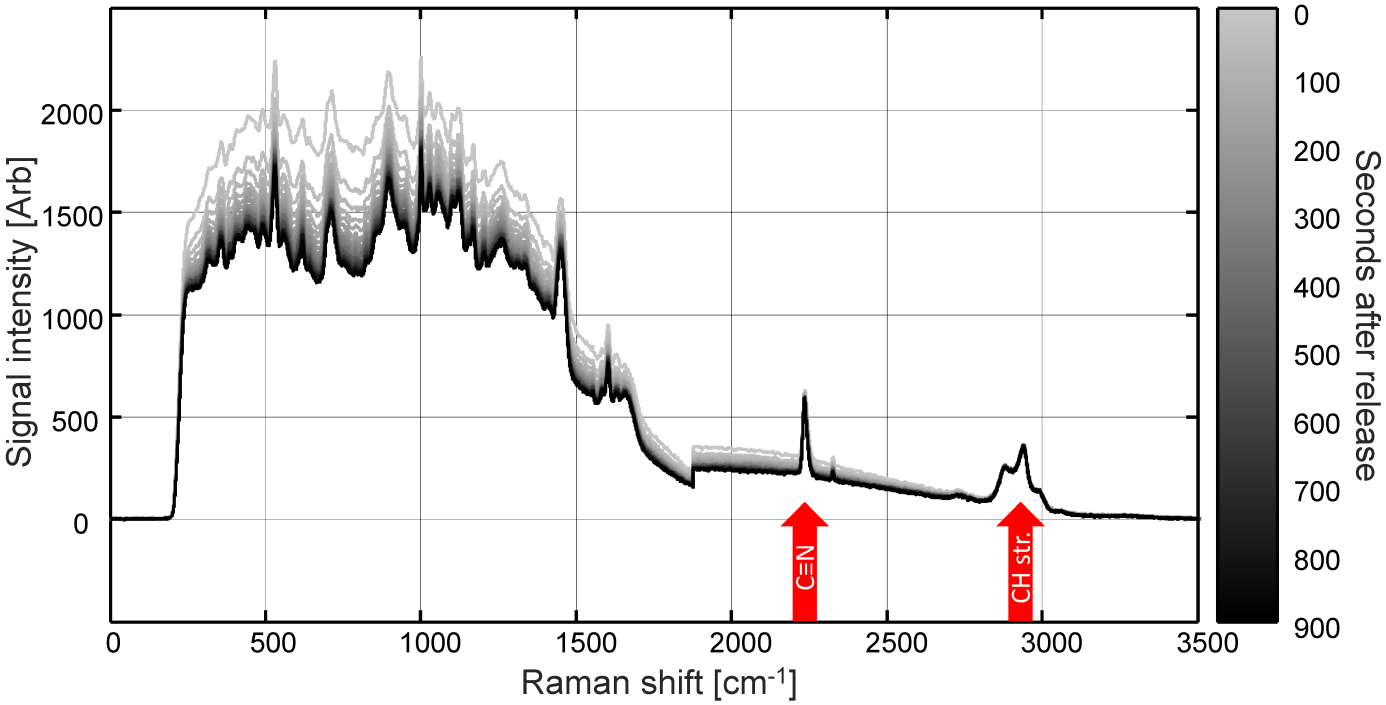
**

**Figure S1. Raman spectroscopy on defence droplets shows that the nitrile group in the CNglcs stays intact over time**. 1 μl defence droplet was measured every 30 sec for 15 min by Raman spectroscopy. CNglcs can be specifically analysed by Raman spectroscopy due to an inherent nitrile group which shows a well-resolved band near 2245 cm^-1^ .The nitrile group in the cyanogenic glucosides of the defence droplets did not change during hardening of the droplet. CH str. – carbon hydrogen stretch, CN – nitrile group in cyanogenic glucosides**.**

**
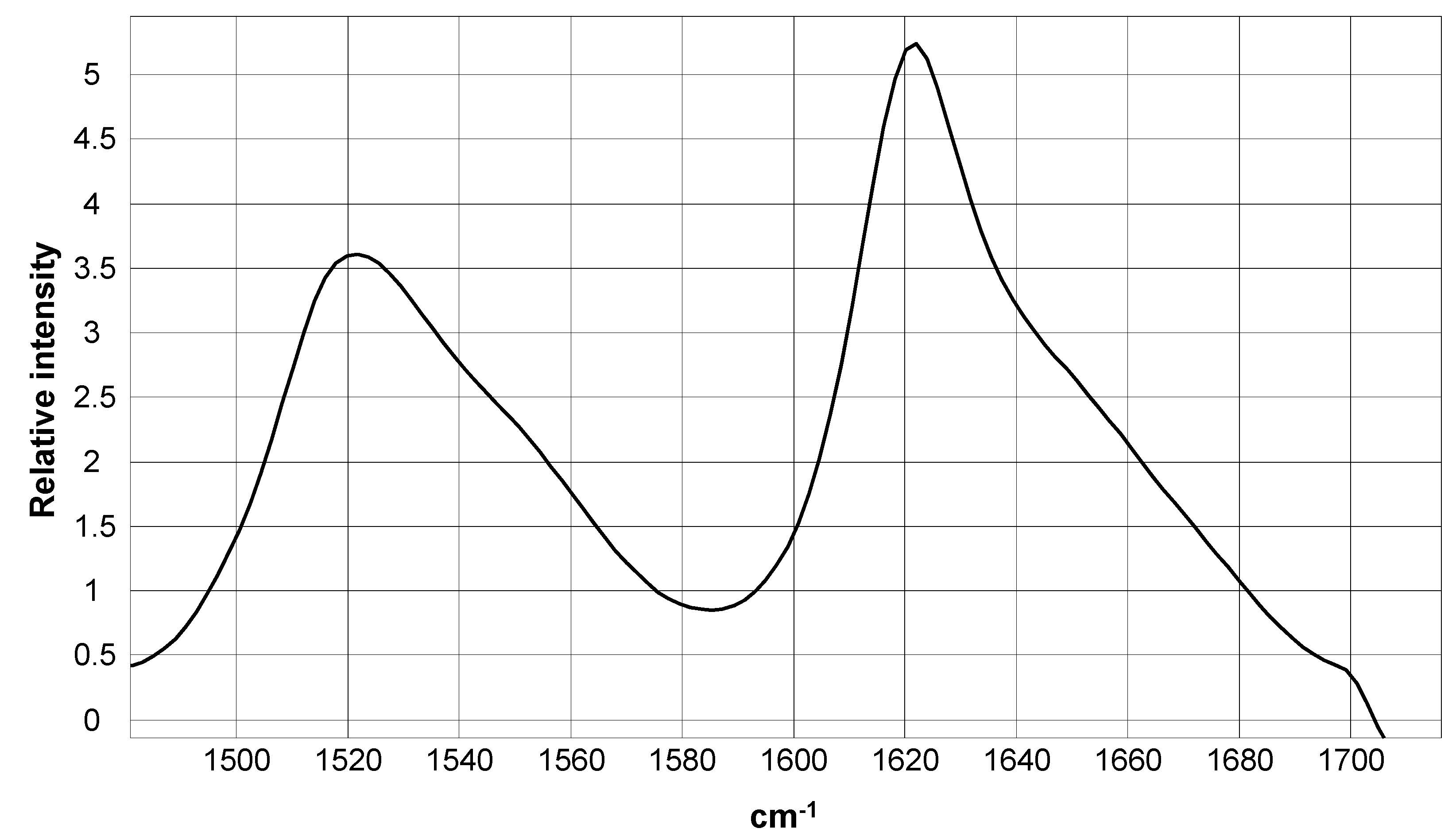
**

**Figure S2. FT-IR spectroscopy on defence droplets precipitated in methanol**. Characteristic amide I and II peaks at 1622 cm^-1^ and 1521 cm^-1^, respectively, are similar to the final state of hardened droplets (see Fig. 6).

**Supplementary tables**

**Table S1. Small and non-proteinaceous metabolites in defence droplets and haemolymph identified via GC-MS.** Metabolites were sorted after abundance and grouped into classes. *β-Cyano-L-alanine identified by authentic standard. Rel. % - Relative percentage, **±** relative standard deviation in %. Relative ratios of metabolites within samples were calculated based on mean value of each metabolite calculated from three biological replicates. nTMS - number of trimethylsilyl functional groups that have replaced labile protons. Note that defence droplets and haemolymph samples were only trimethylsilylated without a prior methoximation step which resulted in multiple peaks derived from reducing sugars. For calculation, these multiple peaks were combined.

| **Class** | **Defence droplets** | | | # | **Class** | **Haemolymph** | | |
| --- | --- | --- | --- | --- | --- | --- | --- | --- |
|  | **Metabolite** | **Rel.%** | **±** |  |  | **Metabolite** | **Rel.%** | **±** |
| **Carbohydrates** | Glucose-5TMS | 93.51 | 7.9 | 1 | **Carbohydrates** | Allose-5TMS | 22.00 | 11.4 |
|  | Myo-inositol-6TMS | 0.98 | 92.5 | 2 |  | Trehalose-8TMS | 18.70 | 46.5 |
|  | Methyl-glucoside-4TMS | 0.41 | 39.1 | 3 |  | Turanose-8TMS | 4.49 | 16.9 |
|  | Pinitol-5TMS | 0.66 | 88.9 | 4 |  | 1,5-Anhydro-sorbitol-4TMS | 1.61 | 47.3 |
|  | Galactose-5TMS | 0.19 | 19.7 | 5 |  | Glucose-5TMS | 1.28 | 68.4 |
|  | Xylitol-5TMS | 0.14 | 119.6 | 6 |  | α-Methyl-glucoside-4TMS | 1.00 | 10.3 |
|  | Maltose-8TMS | 0.14 | 114.4 | 7 |  | 2-Deoxygalactose-4TMS | 0.76 | 98.5 |
|  | Fructose-5TMS | 0.05 | 90.7 | 8 |  | Methyl-gluco-furanose-4TMS | 0.61 | 15.0 |
|  | Methyl-xylipyranoside-3TMS | 0.03 | 19.4 | 9 |  | Maltose-8TMS | 0.33 | 90.5 |
|  | Rhamnose-4TMS | 0.02 | 59.5 | 10 |  | Xylose-4TMS | 0.25 | 6.3 |
|  | Ribofuranose-4TMS | 0.02 | 16.3 | 11 |  | Talose-4TMS | 0.24 | 16.8 |
|  | Lactose-8TMS | 0.02 | 64.3 | 12 |  | D-Glycero-D-gulo-Heptose, O-methyloxime-6TMS | 0.18 | 27.7 |
|  | Sorbose-5TMS | 0.02 | 32.4 | 13 |  | Mannobiose-8TMS | 0.14 | 5.6 |
|  | Ribose-4TMS | 0.01 | 69.6 | 14 |  | Arabino-hexos-2-ulose, dimethyl acetal-4TMS | 0.14 | 20.3 |
|  | Arabitol-5TMS | 0.01 | 64.6 | 15 |  | Talose-5TMS | 0.03 | 52.6 |
|  | Pentitol-4TMS | 0.01 | 58.5 | 16 | **Amino acids** | Serine-2TMS | 8.35 | 11.5 |
|  | Xylose-4TMS | 0.01 | 64.6 | 17 |  | Pyroglutamic acid-2TMS | 6.05 | 11.1 |
|  | Arabinose-4TMS | 0.01 | 33.5 | 18 |  | Valine-2TMS | 3.02 | 15.7 |
| **Amino acids** | β-Cyano-L-alanine-nTMS | 0.97 | 140.0 | 20 |  | Serine-3TMS | 2.87 | 47.4 |
|  | Norvaline-2TMS | 0.02 | 46.8 | 21 |  | Glycine-2TMS | 1.14 | 96.9 |
|  | Threonine-2TMS | 0.02 | 43.0 | 22 |  | Glycine-3TMS | 1.01 | 80.2 |
|  | Serine-3TMS | 0.01 | 49.6 | 23 |  | Alanine-2TMS | 0.95 | 8.3 |
| **Organic acids** | p-Coumaric acid-2TMS | 0.90 | 6.7 | 24 |  | β-Cyano-L-alanine-nTMS | 0.89 | 54.2 |
|  | Glucaric acid-4TMS | 0.09 | 25.6 | 25 |  | Threonine-2TMS | 0.54 | 79.6 |
|  | Fumaric acid-2TMS | 0.04 | 73.9 | 26 |  | Proline-1TMS | 0.53 | 21.6 |
|  | Stearic acid-1TMS | 0.03 | 9.0 | 27 |  | Isoleucine-2TMS | 0.37 | 24.0 |
|  | 2,5-Di-hydroxy-2-methyl-propanoic acid-3TMS | 0.01 | 62.9 | 28 |  | Phenylalanine-1TMS | 0.35 | 4.5 |
|  | Citric acid-4TMS | 0.01 | 124.8 | 29 |  | Leucine-1TMS | 0.25 | 19.9 |
| **Ste-rol** | β-Sitosterol-1TMS | 0.02 | 27.6 | 30 |  | Proline-2TMS | 0.11 | 47.3 |
| **Unknowns** | a | 0.06 | 64.9 | 31 |  | Tyrosine-2TMS | 0.04 | 24 |
|  | b | 0.01 | 16.8 | 32 | **Organic acids** | Citric acid-4TMS | 2.55 | 32.0 |
|  | c | 0.02 | 29.8 | 33 |  | Malic acid-3TMS | 1.91 | 55.5 |
|  | d | 0.01 | 38.2 | 34 |  | Succinic acid-2TMS | 1.63 | 21.2 |
|  | e | 0.01 | 32.3 | 35 |  | Oleic acid-1TMS | 1.40 | 57.4 |
|  | f | 0.01 | 4.3 | 36 |  | Aminomalonic acid-3TMS | 1.39 | 106.4 |
|  | g | 0.02 | 11.8 | 37 |  | Stearic acid-1TMS | 0.70 | 70.7 |
|  | h | 0.01 | 44.5 | 38 |  | Phosphoric acid, 2,3-bis(trimethyl-siloxy)propyl-4TMS | 0.70 | 114.2 |
|  | i | 0.03 | 30.0 | 39 |  | Pentanedioic acid, 2-(methoxy-imino)-2TMS | 0.67 | 10.3 |
|  | j | 0.01 | 91.9 | 40 |  | Methylmaleic acid-2TMS | 0.43 | 38.6 |
|  | k | 0.04 | 23.1 | 41 |  | p-Coumaric acid-2TMS | 0.33 | 13.8 |
|  | l | 0.01 | 8.3 | 42 |  | m-Coumaric acid-2TMS | 0.32 | 33.0 |
|  | m | 0.10 | 71.0 | 43 |  | Threonic acid-4TMS | 0.26 | 24.4 |
|  | n | 0.52 | 45.1 | 44 |  | Linoleic acid-1TMS | 0.26 | 53.6 |
|  | o | 0.38 | 50.8 | 45 |  | Itaconic acid-2TMS | 0.25 | 10.1 |
|  |  |  |  | 46 |  | Cis-Aconitic acid-3TMS | 0.19 | 80.5 |
|  |  |  |  | 47 |  | Myristic acid, 2-(1-octade-cenyl-oxyethylester, (Z) | 0.16 | 77.6 |
|  |  |  |  | 48 |  | 3-Methyl-3-hydroxy-glutaric acid-3TMS | 0.14 | 25.5 |
|  |  |  |  | 49 |  | 2-Ketoglutaric acid-3TMS | 0.08 | 26.0 |
|  |  |  |  | 50 |  | Fumaric acid-2TMS | 0.08 | 54.0 |
|  |  |  |  | 51 |  | Deoxycholic acid-2TMS | 0.06 | 31.3 |
|  |  |  |  | 52 | **Sterols** | Cholesterol-1TMS | 1.62 | 88.5 |
|  |  |  |  | 53 |  | β-Sitosterol-1TMS | 1.33 | 76.9 |
|  |  |  |  | 54 |  | α-Tocopherol-1TMS | 0.18 | 20.1 |
|  |  |  |  | 55 |  | Campesterol-1TMS | 0.10 | 44.9 |
|  |  |  |  | 56 |  | Stigmasterol-1TMS | 0.06 | 30.5 |
|  |  |  |  | 57 | **Alkanes** | Tetratriacontane | 0.18 | 10.9 |
|  |  |  |  | 58 |  | 11-Decyl-Tetracosane | 0.13 | 26.7 |
|  |  |  |  | 59 |  | 11-Methyl-nonacosane | 0.12 | 4.7 |
|  |  |  |  | 60 | **Ter-penes** | β-Amyrin-1TMS | 0.11 | 46.8 |
|  |  |  |  | 61 |  | α-Amyrin-1TMS | 0.16 | 62.8 |
|  |  |  |  | 62 | **Nucle-oside** | 5-Methyluridine-3TMS | 0.22 | 65.4 |
|  |  |  |  | 63 | **Unknowns** | a | 1.98 | 86.7 |
|  |  |  |  | 64 |  | b | 0.20 | 30.5 |
|  |  |  |  | 65 |  | c | 0.14 | 3.8 |
|  |  |  |  | 66 |  | d | 0.27 | 68.2 |
|  |  |  |  | 67 |  | e | 0.37 | 85.2 |
|  |  |  |  | 68 |  | f | 0.35 | 98.0 |
|  |  |  |  | 69 |  | g | 0.36 | 86.4 |
|  |  |  |  | 70 |  | h | 0.28 | 88.8 |

**Table S2. Primer sequences used in this study.** *Restriction sites underlined. ** 6xHIS-tag in italics.

| **Primer** | **5‘-3’ - sequence** |
| --- | --- |
| ZfBGD1GSP | CCGCTATATCTCCATTGGAACCGTCCTC |
| ZfBGD1NGSP | CCTTTGCCTCCTTCGTTCCACGCT |
| ZfBGD1orfF | ATGCTTTACTCGCGTAGAGT |
| ZfBGD1orfR | TTATGAATATTCCTTAAAAAGATC |
| ZfBGD1Fxma* | TAAACCCGGGATGCTTTACTCGCGTAGAGT |
| ZfBGD1RHISnot** | AAAGCGGCCGCTCA*GTGATGATGATGATGATG*TGAATATTCCTTAAAAAGATC |
| 18S-Bf3 | ggtgttttcatcaatcaag |
| 18S-Cr2 | cgccggtccctctaagaag |

**Table S3. Amino acid sequences of the proteins identified via LC-MS/MS in the defence droplets of *Z. filipendulae* larvae.** Values in () indicate protein identification scores according to the MASCOT score. Values in [] indicate the protein mass in dalton.

**poly(U)-specific endoribonuclease**  (267) [37033] SHLVPNAIVRSGTSAEITSASQSRWTSY_NHV_KMRLTIVFLLCLAACHADDFAHAAGQIFNNILPNIISNQVTHQNGNTAGNTIQQIGSVVSGVVDYSKTKSYEDLLRQAQGATTDEDLLRLSEEMFNADINNAFNFIQVKLQAKTSAISKDDLAPDNLLNVPENVWNGPTIRPFVALFDNYHKNVIRPEFSTPSEETEQVTYINTILATGPIRSLISFLVNKGLTQLNDYNEQVQLFKKIWFTKYARHWTGLCKCSCAFENVFMAELKSDTVLGLHSWLFFAKRELDHKANYLGHIDKLDLSGKGLILKQHSVLSDARSKCSCSRRSG

**acid phosphatase**  (206) [57811] WQSSAATTFKSSLRKTSTTSTPSTTTTTTTTTPAPTTTTEAIEPLKENEAAENPDEELLLAFVLNRHGERTPDEDELSLSNEKEKLQEYIDIDGYEGLINVGKQRVYHLGKFLRERYGPDGLKLLNRLYYHKDIALRSTNKERTKMTAEIAMAAVYPPEPEQQWGEGLGSLWQPIPYTTVPLAEDYLRFYTNCKPYKTLMAKAKVDAIHEELAPYGDLIDLIVEKTGRNFTENPTMFQTLFDLFRSQTGLGLSIPDWAEPIMPRLEEAARLGYKIYFRNDQMKKIAGGVVLNDFVQAAKDIVAGKKVEQRFHMYSSHDFNLGAQIEATKIVERDAIFPEYASLFGLELYKNKVTGKYSVLPIYLPQAGEQNELFHLHFEACEDTTYCDLDKFLTSTRQYLMTEKQFYQTCGIKTELGN_TLCN_FRLSIDFWIASRMLTN_IMFFFVTFCLTVPAVYL_KYCCLVYTYFKIFC_TIFHQNSSIIAYHVLLHCSINILFFIS_KKK

**aldo-keto reductase** (112) [54008] FFFFFFFFF_RGGKAQLGSRQYVNIFREENMCVPVRQLFLFFIAIIGTSASLAPTIDLNDGNKIPVVALGTGRGTAKQNETLDDVHNAVYWAIEAGYRHIDTAAVYKDEEQVGRGIADAIAKGLVTREELFITTKLWSDKHATEQVVPELRRSLQRLGLDYVDLYLIHFPIAIKADGTADDIDYLDTWKGMEEARRLGLTKSIGVSNFNSEQLDRLIANSDNKPAVNQIEVNPTLTQESLVSHCQKLGVAVMAYSPFGFLVARKTPSQVRIDETKLEAIALKYKKNTSQIILRYLLDRGLIVIPKSTNKERIAQNIDLFDFRLTADEVAAVSEYNTNTRVIDASHYKNFPYYPFEKN__IITSCLFICGL_NCYLATFEVV_NV_DESFIFNVPIKNRSKYFVLFLKTLWARWE_EIFA_VHLTNFHVGGIRIYL_VYSKHI_VFISKYHKILTG_PNAII_GL_ALR_LHFNMVRLGLQ_LFIISL

**aldo-keto reductase** (114) [42461] MVKYCFKTVTVIILIALFNDVQSTGVAPRLKLNDDRYMPSFGLGTWLGFTKEGVRIDPTGTEVQNAVEWAIDAGYRHIDTAAIYNTEDQVGRAVNKKIADGVVKREDIFVTTKLWNTDHAKDAVLPALRKSLKTLNLDYVDLYLIHWPISSFANGSLSNVDYLETWQAMIEARDLGLTKSIGVSNFNQQMLDRLMSLSPVKPASLQVELNLNLQQPALLTYCRQHGIIVTGYTPFGSLFHSKATPNAPPPRTDEKALLDIAAKYNKTVPQINLRYLVELGVVPIPKSVTKSRIEQNIQVFDFQLTQQEKDLLKSYDRHYRTIDVKFWKDSPYYPFEK_CFFFWYKFFFFFFNF_KFFFKVIFFFFFGW

**α-amylase** (167) [79765] FFFFHSFTIERKTLDYFTR_NKHLPTLSITYNVLYYLCLSIINK_FIDE_DVTYRLKLHTGFLLFIITYLISRYIICLRSYLYLFLINTYANSSTILVFR_MLY_MVDMRVSSLFTFAAVIILVGAADTEWWRSAAIYQIYPRSFKDSNGDGIGDLNGITSKLGYLKQTGVDAIWLSPIFLSSMYDFGYDITDYRKIAPEYGTMEDFHKLLKDAKEIGIRVILDFVPNHTSNESEWFVKSAKREPGYENYFIWADGKPDPEQPGKTLPPNNWVSVFSHSAWEYNAVRGQYYLHQFAIAQPDLNYRDPRVQQEMKDVLRFWLDQGVSGFRVDAVNFMYEVDPQHFGGKYPDEPLSGGSNTDPNDYSYLDHIYSKNLNETYDVIYDWRDLLDEYTERDGEYKIMMTEVYADLHSILRYYGNDKRNGSVPFNFGFIEEISNRSTARDIKMVVDKWMTYMPNGKVANWVNGNHDQSRLASRLGVDRVDAMNMLALLLPGISLTYQGEELGMTDGYITWKDTKDPQACNTQDPVNYWKKSRDPSRTPYHWDSSAHAGFSSTAGNTWLPVAANYETVNLAAEQNVLRSHYKVSLMK_TKST_KSIFKNLHASF_SKNSKDEA_FQVLSTNMLEFRLYVRSKFAYISWNLFVNLYSQRSVLDIYINSTQWSSPQFLYSRKIIPYVIVICMVPNVSVQCAPA

**α-trehalase** (115) [83156] ALYH_RSPQSYRAVRTRSILLLPITN_KQCRYDITTVDKFIEDIFFYYHKSVAVPES_SSLKNCGKYFKMKLIWLVLAATSAVLAADPPSCRKPIYCDSELLHYVQTARLFPDSKTFVDRQMKNNEKTILSAFEILLNTTNHKPTNEELSDFVNEHFEEGNELEIWTPCDFTTDPEFLKDIRDDSLRLFGKDINNIWPTLGRKVRKEVFRSPDQYSLIPITHGFIIPGGRFKEIYYWDTYWIIEGLLVSGMKETAKGMIENLLELLNDLGHIPNGSRWYYEQRSQPPLLTAMAKLYYNYTKDVQFIEKYISLFELELDYWLDTQIVTFEIDDKSYTMLRYYAPSTGPRPESYFEDFKNAQVFHSNDRQQDFYTDIKSAAESGWDFSSRWFISNSGDNDGNLTNIHTKHIIPVDLNAIYAGALQNVAHFHGLLGNPKRAAHWAYLAKQWRSNIEKVLWHEKDGVWYDYDMQHGKHRQYFYPSNVAPLWIGTVEDRFLRKHTPKIMNYLKVIGALKYPGGVPTSFNYTKEQWDFPNAWPPLVSVVVNALEALDTKESQNAALDVAQTWVRACHKGFQESHQMYEKYNAETPGQVGGEGEYTVQNGFGWSNGVVLEFLAKYGKVMTLVD__VGSVGWVENNDKFCRRVLDSLILICV_R_CCSXXXXXXXXXXXXXXXXXFHPLRG_NSFLMLTYRYVIK_NKLEQYSVINI_NICMYI_LYLTN

**apolipoprotein D** (100) [56304] NKMYRWPVSGFVLLALCGSVFTHTYHLGECPVVEPMPGFQMNGMLGVWYVIQKTSTASHCITYNFTRTNEPGKYELQQISQHFILGLTPLKHEYKYTGELTVPDPAVPARMKVRFPLSVAGSASYTIMATDYTTYAAVFTCQKLAFAHRRSATILSRTKELDRMYVDKMRSKLSSYGVDPYDLSIISQSECPHHPDGKPEGVNININPETFSSHNIGEAVRKTGGVIADGVEYVVETGKKVYHKVASSKEDLTEPPAGPDAVRTMDADAEWLP_TYQAANRSTTLANCVALSFRCCSSVSASIS_LP_ILKTIHDYL_VP_VTAFH_MHMKVYTVLTKWY_PKKINF_IFSVCLFVPPNLRNGWTDFNGTFTGR_LML_GVTQATFYLGILAESLKLTRVKPGAQLEVKK__RKYSYSLFKFSLYCSKFFKENLKTIRNKYILLIISMMYI_VHIYLLIT_NLLLHLY___T__FYGNFF__HRVLDALTATFL_PNLIRLQKHYLRTTRFGHG

**β -glucosidase ZfBGD1**  (104) [69394] QV@S@CAS@V@TMLYSRRVVQSKSVLIGFSFLCMLQGISLQDVHRGYSTYMFPEEFIFGVSTAAYQIEGAWNEGGKGENLWDTYLHKHPNFTEDGSNGDIAADSYHKYKADVQMIKDLGVKYYRMSISWPRILPYGTDNYINQEGVNYYRNVFDELLKANITPVVTLYHWDMPAILSDFGGFSNPVIVDYFEDYARVVFKLFGDVVKMWITINEPHMLCMGGYGLDMFAPALNSSGVGEYLCAHYTLLAHARVYHLYDEKFRPTQKGQIGITLDSFFPEPKNKSSEEDIEAAERYLQMHLGFYAHPIFSKNGDYPSMVRERIDKMSSHQGYSRSXXXXXXXXXXXXXXXXXXXXXXXXXXXXXXXXXXXXXXXXP@HRSKNKP@SKLA@ARSRLARCIPSWFPEVAQVDIEKIWKSRSNNNNRERTLRRRPTQGLRESVIFQRIFESVVTSKI@RRC@CPRILRVDSDGRF@MGLWLQGQIWFISRGF@KPRKNSNSQTISERLQKYRSDAAYPIRLYKGIDNQWNNGSF@GIFITLVNNIIMYVPEICTYLMLQVYILFVICNIIC@LVVLKKKKKPKKKKIFFFPKKI@KNFFFKKKKKKKKKKKKKK

**calcyphosine-like** (149) [25855] KLRLLCLSRGAAGVLGLGRVFRRMDDDGSKQLNKEEFVNGIKETGLEFSSEEAEQLFDKFDTNKDGSISIDEFIRQIRPPMSDSRRAIVELVFKKFDKTGDGIVSVEDIRDVYSVKSHPRYMSGEETAETVLNKFLANFESEGIVDGKVTLEEFMNYYSGISVSIDNDCYFDLMMRQAYKL_TSDNNFIH_HRY_CNTKTPKGG_SCNDSLAYYVFCTFFDLINFSTC

**chemosensory protein** (207) [41248] RGGTIHVGIGKDYTQLVSKKNWKLFMCTVTCVFQLIKRQCAISPMSVANL_SSCRTVNIAIVKMKSFVLFGIVALAACAIAEELYTDKFDNTDVQEILSNKRLLVSYIKCALDQGPCTPEGKELRDKIEDALKTHCAKCTEKQKKRIKEVIYHLIHNEKSYWEELVAKYDPERKFSHEYEKELQELSAK_NSRLSNGFHLYF_NTKKYLLNLLIYSYLFSIYNIKKSYYVFKKSLFILLKSTSCVRYL_TLPFLVNKMIAFGSFYLTQMRDIKSIQNEQ_LKY_VVI_IAS_KLLCFYLLRIKDKGPSVW_AVYGITAIFPQPYISNLTLTQDISEA_YYKENRLEFSMFVC_NYLTDFK

**chitinase-like** (180) [64228] RRCRQTSFTVQSTQWTHVCNEDPGNLCFAGGRRRCRPAHTRQSPLLL_QQKLREGIPSAHAAK_LGPSAVVLHSPLVRVRRHPTRHLQDGAPQREPGRGPRPPELQDHHQLQVQIPXXXXXXXXXXXXXXXXXXXXXXXXXXXXXXXXXLGAEQHGFDGIDLAWQFPRVKPKKIRSTLSSLWHGFKKTFGTTPVDDKETEHREGFTALVRELRAAMNLKPNWQLAVTVLPNVNASIYFDVPSIINLVDIVNLDAFDYYTPERNPKEADYTAPTYTPQNRHERQNIDAAVSYWLSAGAPSTKLVLGIPTYARTWKLDSDSEISGVPPIHTDGAGEAGXXXXXXXXXXXXXXXXXXXXXXXXXXXXXXXXXXWSLGELRRPGHGWTKSRICQI_EPWWSCHRRSFHGRFPWSLHW_QVPDSSRCQIPSVNLRYYRYECEHSTK_RMSFSCIYII_IS_HLYHVQDLQILSIKYDCIIFIIMLRIQHKINYIEPRFN_SSDLRTVEFLAICFSAYYVKKKYYFYTSQFIVTCLGKYNVNKYLH_IFLFYINKTTKICCPKKKK

C-type lectin (166) [11927] AREFRCDYTYYDVADGWLKFHEIPANWYEARLRCHLEGGTLASPVKGIREAMLTLLAAKKVDAPCQLYTGIHSFFSKGDYASIEGVPLRRIPHQWAVGEPDNY

**C-type lectin** (206) [66538] WIVGGGRGMRRCYKGGSRASRAQ_VRVSRVARAHPLKCLLHARTSPQTHE_HSKKRDKMQFALATCACLLVATLGPVAAQRITTIQLDGVQYFISRMNPYSPELNYFLAYQYCRSLGLQLASFETKEKADSITTYLINAGYNKYDFWTSGNNLGTDMYLWMSTGLPFNATFNYMRRIAIDAATQHNDDSMDPLDMPQGSTAPQRTARHGTEHVMTNGCVALKAPGFHWEPQHCGEIKDFICEQTRCYFYNYGSIPVSSAQGXXXXXXXXXXXXXXXXXXXXXXXXXXXXXXXXXXXXXXXXXXXSPHIKTGAVLKAPPQIDSHYSRAADAHAHDHEEETKEEATQGPYEDDGMVGDDPNAYLAHEAREAPIADDNAPSTSEPDATEHSVHASGMLAPPSY_RPTDVIKFSLNMTTKYFTRDNSFHSL_KQLEKIAFFRDKNYLHTLQF_GRRPIFCTTFYSILN_YFS__NKYT_CE_FKVFRGNVVRKRRCWRLVYDNLPPLSYSMLQEKQR_VSKFMYSMIDLWWYAEKQRNMVI__I_IGCDT_IWDISFCN_NSSLR_YL_YVSIISSNVK_EMTRQ_FTEILKK_NSFFLKK

**C-type lectin** (248) [61397] QQCLCDFVCPAVIFIHQIKMRTWLVVACAAALSALSAAQQAGRFLSLPVPEKCANRPKEFFYRGHNYFYSGHVPAHANKKVDWLDARNICREYCMDLISMETQEENNLIFKLIQQNDVPYIWTSGRLCDFKGCESRRDLEPKNVFGWFWSANREKMSPTTQIPNGWGYNPWSQTGHKKQRQPDNAEYDINGTVESCMSVLNNVYNDGIAWHDVACYHEKPFVCEDSEELLNYVASTNRGLRL_FKLTLSITYDPGIIHGWSVNI_ELTPFVNSLDFNEMVTMPLC_SRLRSIHDEYLPGYTLGTVSXXXXXXXXXXXXXXXXXXYLIYLNNRIVYHYVLSMFSTYLIIAYNFII_FIYNNVGEYIPSYSEFLNLIYLWN_RAILTTQCAGSNKHLGRWRNVQASTPSLLDFRLIDSIHSIRDDSKLILLCKMRLRLITVYCSLLNFKTHNKNMKTLAVLAIFYLMSYE_LI_FPLFY_ICMYIFFAVLL_FM_VVI_NKLLSYKRYYLDLFTQWMNLNLYIK_KNCKCK

**cuticular protein tweedle motif** (189) [46712] VRNWSEAPASYLQTYKRERQSKVATLDVKCVSVNVGAFIASP_ELXXXXXXXXXXXXXXXXXXXXXXXXXXXXXXXXXXXXXXXXFNGYLSNDNLLHTAYPTFSSSLSSGYNLGGNSEFNSGYLSGLSETKKYYPISGINPYLTSTKTYFSGSQPSLTYASGTDFGYKSQDQYKISTSNQQTQSTTHQQEYYDIFHKGPPQVYKHVYLHAAPEEPEAPKTRHSIVLPPKQKHYNVIFIKAPTPPAGGSHVLPVQQESEEKTIVYVLTKNPDTVHDVVVPKFTQKPPVKPEVYFVKYDNKEGAHTVIDNIVHDYKGQDYVTLNGQPLASDTGYESISDTTSGLNNFVTTNAAGSINLPETLGSFESTGGSKFTLTSGPGALETVDNSNTDTAYTISSQSSSLTDKDVSLGTNYESSTSTIISSTEAPLI

**cysteine protease** (332) [109902] GAGRGAGGAGGRQLICRRGKIRRRLELLACLRREP_HTPRHSMKRATLLLVCCTLGAAWGSQAPLDPVAEAQPNETRERYRRAADHWLQILSEYAHKYSGEQHVDRPQGFPSPFPVDLPIAGHDGKTVETLPLLVLPLPVPVFQPAAPATECIQTKKEEESTEKKDSHYITTVTIPKPEPELITPVRIDETPSKSDSSEFVTPIKINPTTGPSPSDAYSPPKSDSYYINTVSIPKPELELLTPIKINPTTGPPLTTYTPSKSKFTTPNLPPKTQYTLSPSTYTTYKPQYTTSKPQYKPIKAQYASSTLNFSSKPQYPSPALINQQLVSKYPPATTFEKPSPYPLPTTQYPASAPQTPEGVSQYVETASRFSETASKQPAQQYTESTQQYPKPTQQYPQPAQQHNEPTQQYPQPAQQYPEPIQQYPQPAQKYPEPAQQYPQPAQEYVELTQQNLEPAQQYVELTQQNLEPAQPYSEPAQQYPEPTQQYLEQSQYLESAPQQQESTQRYPSPVQQFSEPAQQYPKPAQQYSEPAQQYPAPISQYNKPTQQYPESAPQYQQSSLEFQAPIVTETYEGYSTYSLVEMYRQQSPAAPELPAVVLSEAGLRPQPAATPARPSPFGSTPRPGQYVPTNPFGSLSYNRNPPLPRFDRPMQLRAELSVPQAGYTEPYTLWWSPTNKAARVDFHGGATSTFLIILPNRSVQRIVVRLDRSGDAGDVRRCGMTPPQTLPFAELEALQRYPPALPDLKLFSFAGYTTTDGGRTERWQYETSGKAGELGAARGEKLTFHHELQLTRAADGVSALPQLYTVTVDSSVLGARFHSYSHRYSEVRYQPLAADLFRVDLNATCDALESLDPAQPQHAARLDPLREFTRPARDPRYDALFDRFKNEFNRKYADDTEEAVRKALLIQKIQYITSGNREGATFEMGVNFLSDRLDAELQVLLGAVPEPEGIPAEPFPVASSQLGQLAARLPDEFDWRPLGA

**γ-interferon inducible lysosomal thiol reductase** (102) [36485] _ILYNRALTQTSICQSGLQ_SHTIYYMSINLAYIKFLV_YIRKMKGFQFSFVLCTILFGVVIASPRDTVADEVEFQNFTEINENLSPERKEETRKDVVKIQMYYESLCPTSRRFYANVLTEIVQKFGERVEIITYPYAEAETYESAGHYEFKCQHGPLECYGNKLHACAIDELKNMTATVLLTGCMMDRRLQRKGSNDLSADNCGKMLNIDSEPIKDCAKNDRGSNLLKYYGDETKKITLETIPYILINGVVNDRHNFIHDVCTEFENPPAACTEYL_WK_I_L_FVLYFHFYTF_IYLKKFLNYYLKKKKKKKK

**haemolymph juvenile hormone binding protein** (119) [35837] AIMYSDLVVSTRINMFALKLIFCVTVCVIGSRASQLPDFIKPCSRSLPKKELTNCITTQSRAAQSYFAKGMPQYDMPSLDPLHLNEAVRVEAGTFTIKTTNAVLKGLGNVLIKEVEWDLEKLKGHLILFGNMTMVGDYEVDGRILLVPLQGTGKLTVHMYNTEIKINFKLRTNKGKDDVDHREAFDIKATQNFGSAKFRLTNLLGGGVIGENINALLNENYRDIIEEVGPAFVKTIATKVNEIVNTFYSHIPVDELLE__TKKNPYKKS_L_NKIALLINMYNTLYRIW_KKLLSMFTDIFL_KVVNKNLCCVTN_LFYCL

**inter-α-trypsin inhibitor** (981) [114272] EST_VPYPEVSSAPLSAAKR_R_LVRDQRPIQVSPRLTTAAHV_TSFTTRKSQRSPSLRCTLYVERARAPPAIGAIISLSINKPLTKNNNGEEMXXXXXXXXXXXXXXXATEDGNFVVTQSDATSSTVASSSDVTTEEPPSPIKITEMDVLSEVNMRYSHTSVTTRVRNPAKRAQEATFRVLLPETAFISGFSMTIDGKTYTAYVKEKEEAKNIYNQAVSQGVAAAHVAAKARDSNHFTMSMNIEPSSTVFFNLTYEELLVRRNGVYNHAINLHPGAIVPKLTVTVHIKEDQKLTVLRVPEVRTGNEIDATDSDPQNSRAIIEKAAEAREATIKFTPDLEEQKRLMEIYAQKTKESQLQSHLHRWYDDDQEQEPVEGVLGQFVVQYDVDRPKNGEILVNDGYFVHFFVPTDLPPLNKHVVFVLDTSGSMMDRKIIQLREAMQTILSELNRGDYFSIVEFASSVKVHDLKEADVTDDDRRHNYLYYESQSDTTLVPPFQATPENIAKAKIIVGRLNASGGTNIASALDTAVELIQKGIGWTQPATSNETKVEENTETVASNDSPKDVTVKPQEVETSLASKDGAIKLEPIIIFLTDGDPTVGETDPKRIITHLSEKNYGENKATIFSLAFGEDADRKFLRKLSLRNEGFMRHIYEAADAALQLRAFYRQVSSPLLSHVKFVYSPDQVKEDSVTKTKFRAIYAGGEAVIAGRIGDDVTEISPEVLGFCGVDDGLTRKKYEVRSKVPVTRVKKDYLPLERLWAYLTIKQLLDERDAAEIADRPKEENGPETKALNLALKYEFVTPLTSLVVVKPNATKAVNAESVDNKDSGTPILPMSFYNSAAQSAPAPASFGLPGVPGQAAHAYMPISLXXXXXXXXXXXXXXXXXXXXXXXNIIHNHTFNPDDLQALFDHRRTKSPSNVQLARFHVGEGATQCQR_RTGPASQFQRHRTDTLEAL_RD_YSVGA_VTVGRRGVPEGNGRRAGHVRVPRALRRRHTHHR_TLQQHVLPRQQ_VRRSMLSEDDLNFRSCSISIK_KKK

**juvenile hormone binding protein** (83) [33622] _SNVYKVFRLTSVRSFKKT_F_NSIMIRLLLFKMLFIAQLSIMHAAVPPVQKCSLSDSECIKSSAQAFVPLISGGIPELGIEPLDVMKVDKIKIDLAGLNLAVKDIDAKGLKKSVIESIKLDMAKKHLRVVLHTDMTVKGKYKASGMLLILPISGDGDLTIKLKGIQLDMTFPYDVIKNENGKDVIDLKSVTFKHDVKGGVHFDLTNLFNGNKELSTAMLTFMNENWPALSQEFGGPMLQKPVHKIFEAIKIYLQSQPLEETTV_GLIIHTLMYS_LPYGYFVHKLTSFVIKLKFRWQQKKKK

**lipase** (100) [45474] ILGHCIIGKLKLVKMTVLLSFLLLYLSLSRALTTERRSVVADASSKVTDTTLGLAGSLGKGIQKEIAAVKQPIEETLVYIGSSQCSHVKKILGVAYEQIEGEKEPDLNQLTLEYSDRSVRVIYNITAAAHDITQAREFDPERKLFIFVHGFTDDPTQSGFRNISEALMASGEYQVLALDGSSLIYWLYLRATTYVRFMGEKLGETLAAMVSRGVNPANIHIIGHSLGAHISGFTGKTFANLTGKRVSRISGLDPAGPCFSHVDPELRLKSTDADFVDVIHTDGGVYGLKDPIGHVDFYPNSGSEQPNCFYQTCSHSRAWLYYAESVVNPNAFPARRCRSWEDFKKGRCESNISYMGYPCQPTTRGQYFLQTGPESPFGLGRKGLTFQNNEGIIKNIASTIFGR_CFFF

**metalloprotease** (90) [69576] AEWAYTSNITKQNEENKIKVQLELSKQEKQSWEETKMYKWQDFQDFTLRRMFKKYSLLGASALPDDKYKLLIQSVSGMESNYATSKICAYKNATKCDLALEPEITEIFAKSQDPEELKHTWVEWHRLAGARARDNFTQYVQLDNEAAKLNNFKDVAEWWQSEYEVSDFEEQLAKLWEDVKPLYQQLHAYVRKRLRDRYGDHVVSARGPIPAHLLGNMWAQTWNNIESFTRPYPDKVEIDVTQAMLDKKFTPLIMFQKADEFFRSLNLTAMPELFWNNSIIEKPTDRDIVCHASAWDFYNGNDFRIKXXXXXXXXXXXXXXXXXXXXXXXXXXXXXXXXXXXPGFHEAVGDTIALSVSSPKHLRRVGLINGEVEDEQTEINQLYKMGIDKIVFLPFAYTLDLFRYGVFRGTTPPEDYNXXXXXXXXXXXXXXXXXXXXXXMYQLMWSTQGTTCLSSFSSNSTELCANWRESTCLKTPTRNWSIVISTRALLQETL@RAC@KWVRPDPGRMRWRLSRASVR@RPTASWNISSLFTNGWSLRINAPESLLDGSLVKCSIVRQNRKLHWMLKKRQRLNKQQLRSPNRRRRMRRDESK@YLIKKF@NYYYYITLLFIRQASV@FLIHIFLINDT@KK

**metalloprotease** (239) [139213] ASIQ_NQVSSPRLRSYQ_LAETPRSWWSGSRDSQHSSQHGAPSLAPLLGACLSFATPAPNTAPDACHPRKFRYNNIPTPASMSGDAEYMTTNYNMRNNVKLSFWGQRTKLEKRLILVAGVTGVLAIAFLSAFIASLVLRPVTTISDMPQTSELRLSSPAPPAIISKGDINLKICNSPGCIHTASKLLLNMDDDVDPCDNFYDFACGSFLKNTRIPDDKTSINTFSIITDQLQEQIRALLDEPITPEEPKPFVLAKKLYQACMNRTAIEARGVQPLLDMLRRLGGWPVLDGDHWDEKSFAWEQSVYKFRAAGYSVDYFIDFSISVDVKNSTKRIIDLDQASLGLSREYLNRGINDKLVQAYYEYMVDIAVLLGAERNRAKADLYESLQFEIKLANISLPLEKRRNATSLYNPMTISQLQEKFPKIPWLDYINQLLAPHVKVTPDEVTIVNVPKYIEDLEMLLEKTPKRVLANYVMWRVAGASVSYLTDDLRRRQLAYVTALSGKTERESRWKECADTTSVSMSIAVGALYIRKYFDEDSKANALEMVNDIRQQFRNTLTKVDWMDEATRSAALEKADAMSAHIAYPSEMLDNEKLTEFYNGLEMSSDQLMESVLNLTLFGTEYLFKKLRDPVNKTDWVTHGRPAVVNAYYSSIENSIQFPAGILQGAFFSAERPAYMNYGAIGFVIGHEITHGFDDQGRQFDKNGNLVDWWQEITKQKYLEKAKCIIDQYSNYTVKEVGMKLNGVNTQGENIADNGGIKEAYYAYEAWTNRHGEEYRLPGLEKYSPRQLFWLSAANTWCAVYRNEAIKLRITTGFHSPGRFRVIGPLSNMEEFANDFKCPAGAEMNPTKKCKVW_Y_VPATTQAHVTGWYIHYHDVKGPAESQNELTYYLK_NNYL_KVT_QQTLLLV_WSVTEEAKYRNASVVPLQYRCRSRCLRGTFYCTVTEVRHLVGRLVRECVV_MLTASLGS_LLVCK___IIIVSNSESLKM_YLHMARSLYRYYLNFFLLD_AQIILT_YIRVKQEPVFFLLSKSLFLIKIV_LFIFYYLQSDLYVMLDKNFNGN__NYFFIINCGQHN_CTYLIKILFIS_L___VYMYLMILELLELIVLLPTGFQDPIGLMIFMIDFMIY_FYILLLYKRRYFSIVLHDYRSS_IRSLVYNFEQR_SLNGRRDS_SCSDSVSNIAVYCIESHSMEFETSSDILVLLVYLSN_VYIFGYDRIVVNH_NTNIINFYV

**odorant binding protein** (2736) [55721] D_DITYAVPIRSRSE_SIFFLLEESVREPLYKMTKLIVFLVNLFVIFHLSLGALPIIPPFTKCVLTDSECVKASCKQVVPLFFRGSPELNITRYAPLKLKLMDLCTPTLNLVLSDVTLSGLETSQIVSLEPTSDGGMLGKLVCDLKIEGKYEMDGSFMSVPVDSEGNLKLVLNQLFLTGIVKLEEVIQEDKKPHWRFGKHKHHVELLGVPEVEFESVFDGIPRIESPLCELVSKNPKMVAQEMIELIMKFVYEIMIADVNKFFAYLPVQDLIVQ_H_NIK_LFKLKNKCF_IIVNIQTEKNFKKRFHYSVTLLLSLPFNF__TLNIFNVSTRILLFLTDFQKRRRFSIRLRFVCYAITYSKTAGSIWIFFERFH_HEVRI_RWDLGKVERTLQILWAHL_RFGYFL__IVHLISKVKTIDGHWNY_IISTCYPQIPAGTPK_VLMKPSTTYIWQNQFSMPERNWF_AIRPTFLLYFIL_LDNNKEYLI_KKK

**odorant binding protein** (465) [62912] LYEV@LYSVGTLLLGY@CLTLTFKIII@NQF@ENVELHVCGSFRCCRFADQC@PSATTDNEMQSRRRGLFERERNKE@GVVQRWST@IQRDANGPCCLLAHRCQLSKLEAHIDRXXXXXXXXXXXXXXXXXXXXXXXXXXXXXADGQYEMDGHLLVLPIQGKGKIHAKIKKIDIDGEANMVEYTAKDGKKHWKITTWTHSFELKDKAEVKFDHLFNGNDGALTKAVRELVRDNPNDVVNEIAPNVVEAMIGRLAENVSRFFKAFTIEQLALD@PLKFLYRDVIY@ISAFILFGLGNLCMYSA@VSLXXXXXXXXXXXXXXXXXXXXXXXXXXXXXXXXXXXXXXXXXXXXXX@HVE@@CYCM@@YLGVKY@TISIHL@FVIFISFLLCTAI@DSLRLNKYRTIMES@QYQNLKN@KKISTFFIID@FLP@KIQ@SL@LHFINYIIWSLKCHR@QNTARWYHNKQ@KNNYFSSSHEVGAYKNVNAFMNI@NIWLS@TSVYLETKGMQ@L@TVELKRRII@FNSKFNQITQFFIIIISRRFRVNSCCCIGRG@CKSA@LALDKTAYVTNPSGRK

**ommochrome-binding protein-like** (60) [38698] RFLSVSNHQSIFVSQSGRFYAVVITTTMKLLLFISCLVISQATVIRKPDQRECTGLPINEALYDKEILKEGLERPYLLAVDRSNDILYFSYSLSTNDDQFKSAHIDLKTKQFENIDSIVNGFAQTVDQKNHEVYIGSSDGIYKYDYLKRSSVYLAEKGKDIWNVYFNGILYFSEFPSEFMYTYQDGRVSRFKELEDTKADHMVIDNDGDIIFSNATGLFTQKMGTKDAVYFPLSEYVRGLTTDINGVPYVVRYNGISIVDKASHTLKKVLDLEDPFGVTFDANNNMIYSERKILIRLKPNKNNNCA__VITHSIRYVYLNLLYVFFINK_VYFYGEK

**peptidyl-prolyl cis-trans isomerase** (86) [11560] GTLDDGTKFDSSYDRDQPFTFALGNGQVIKGWDQGLLDMCVGEKRKLTIPSSLGYGERGAGNVIPPHATLHFEVELINIGDSPPTTNVFKEIDADKDQQLSREEV

**peptidyl-prolyl cis-trans isomerase** (221) [36884] WSQVELLELRSSLPS_FVIYPFYFALQFQQYYT_VTNSKSIP_RLATLKRVWTLII_S_IYCNI_NSNCT_VKITRKKIKLFVIMGALATGIGLMLLLATAQSDEAPKGPKVTHKVSFDITVGGQPLGTVVIGLFGKTVPKTVENFYQLALKPQGEGYKGSKFHRVINNFMIQGGDFTKGDGTGGRSIYGDKFQDENFKLHHYGAGWLSMANAGKDTNGSQFFITTTKTPWLDGRHVVFGKVLEGMEFIRKIEQTPTGANDRPVQDVVIADSRAEIVSEPFSVTKESAQ_HKSYIAI_FS_DISHHAFLGNVSYKMILKINYVFFEIYFYYNKKKKK

**peroxidase** (197) [80802] FFFKGNFYFCN__KPNMAKSIKAYLVLLVYVCSTFANGYNHLLSYPAQAFLQMVGNQPAQTHGSVFSSILSPEFPQSRMPVNGFGRWMGMPSQRGRTDQSFSNPPPPQGSGLSPAEQSCGITPPFCTNTRYRSIDGSCNNLVKPTWGIAQSPYGRLLPANYGDGVDAFPRSSSGQPLANPRDISLRLYPDRQLVDPIFNLNGQQWGQIVTHDMSLTAGVTQSHREPLVCCDDNGQLSPDASTNPSCAPILVPPTDPVHAPQGTTCMNFVRTSTTRDRGCTSPNAPAQPISTVTAYMDLSLVYGNSMAQANPIRAGQGGRLLTLVRAGKEWPPQDPNVTVTCESAQSPNEPCYLTGDIRVNQNSQLTVLQVILLREHNRIADSLALLNPQWNDETLFQEARRIHIAEIQHINYYEYLPILLGVENMIKNKLIYPGVQGYVNDYNPSVDPSILVEHATAAFRHFHTLIRGYLQLITESRVMAGAIRMSDWFNRPLVLELNNGNSFDNLARGLTVQQQEFSDQFFDSEITQFLFKRNNTFGGDLRATDIQRGRDHGLGTYVAARKVCGLPVPQTFQDMLDYISQENIQILQELYESPEDVELVVAGVLERNVPGAQAGPTYLCILTEQFYRTRVGDRYFYENGADPVTAFTPSQLSSIRHGASMARLLCDNVESIRLMQPKAFLQIKQSNQLVPCEQIPEIDLRLWQDTLGLFANK_VLRKIH

**pheromone-binding** (89) [35513] ACLLFL_LCMVG_KYPNTSRYI_MVLLWSLH_SSHVNIVGCL_G_LTIALFIRTE_EISVSV_RCFVR_TQALFVYFAEQSGAMKAVLVLIGLVAIAFAADKYNSKYDNFDVDSLLNNDRLLKAYLNCFLDLGRCTPEAADFKRALPEAIATVCKKCTEKQSSYIKKVGKHTIAKHGDLWDQIVKKNDPEGKXXXXXXXTSSLTAKSLRNNYL_LKSIFNLYFITILFVSNTP_LPHTL__S_RDL_KL_LKFLEFY_LTIKHDNLTFKDQSEIISMKEKVHLTYLADQIIVYVSSLFK_YIIILTDMFVYL_NSNKHIKKHIKKK

**phosphatase** (64) [43873] ITDNMLSTFTRKAWPVWRQKLGDLTSKGELLEGFIGEYFAAWLRETRLLPGGCPSEDLVRVRANVMQRTIASARALVKKAFPECNITVFHEQNYDPVFNMGVLNETDEFKQEVSKELNDFSRGLQLSDAFKELQEILGYKDSDACKKFGRCSLVAEETATDGRIRVGEVLNEFEPLEIGKIIVDTFMMQYYEGWPIENVAWGLVDTEEKWRKLTSIITANLNLCFNITRLAQDITRPTVKYIKDILLNEKELPKITFLYGHDANIFLLTNAMRFEPYNLPGQFEKTPVGGKIVFQKWYDEDNDIDLLKIEYFYLSWNQIRNGTRLDDENRLKIHLLKLRDCKVDDKGFCPWMDFIKLLKSL_D___NNNRSN_SALNVNI

**phosphatidylethanolamine-binding protein** (88) [38397] FFFKKFFKKKKIFFYKKFKKKNPLFFFKKKKKNLLLFRI_IKLENMTTMGRILRCAIVLLIADSTMVNFRVAARAMATVAKSFQTHGVVPDVVPNPPSGYVTVKYPSGAEVKEGNELTPTQVKDMPKVSWDATPNQLYTLAMTDPDAPSRKDPKFREWHHWLVGNIPGNNVAAGETLSGYIGSGPPQGTGIHRYVYLVFKQPRKLEFDEPRLSNKSVDGRAQFSTKMFAKKYNLGAPVAGNFYRAQFDDYVPLLYKSLGA_YLL__TVIFTM_F_LLYKSL_LTYRNTQKEEERKKDAA_VKSGNQSN_TMWLLYSSILSIL__KSRIRDSVH_KFLTKIDRGQ_TNS

**plexin domain-containing protein** (55) [132789] QITAQRDPSNLKYENHRYYKSTFISEPNYFDQHWALITKSKPIAQPGRKNTRRRDRTVTLSFGFPIHGRIVKTITITNGGFIGLGSTDGEQESSQYIAPLMAPFDNRTSNQNGIDLYDDGEKFTAVWRNVNLQDDRSKLFTFAATLYKNGDIIFAYKDIPVELQSISNGDYNVGIQDSYLEDQSSATLSFKNYGITSGTILKLIAIPFENKHPEESKPSNTLPTTQITRNLLKGEHDISNLKYQTYSFYKIAFIDNIDYFNQYWNSIFKLRSLRTNWRRVSNLYETDIELNFEFPFYGRFLRNFKISSGGFITFGDSTHYISPLTANFEIASNIEDAVKLYEDDEKFITVWDNLYLKGDRSKLFSFAVVLYRTGEIVYLYKYIPYSSQTLFDENGVIRIGFSDGDDESRLILFEDYDISTGTMFKILSLQSYSTEPQIKPRPSVAPQKPAKLIQAEEDVTNLKYQNYGSYQSVLVGNLEAFNDYWGAIFESQPLKKIWRPNSYRYETTVDLSFEFYFYGQPIRRLIVTSGGYILLGDDIQDGHAKRHYLIPLLADFEDATSAQKVKLFDDGEKFTAIWEDVTLRTDKSKSFTFGVILYKTGEIIYIYKYLPLSIQELYEKDDQVRVGFTELSNDETETLQQVSFNNYDXXXXXXXXXXXXXXXXXXXXXXXXXXXXXXXXXXLICNTMPIVISKLL_LIMWNISTNFG__YSDRIPSE_VGAKA_SFMKQPLISALIL_VTDASSLMFKFQAVVSSSCKTKIRELKNT_PL_KLILIYQMI_KNL_NSMMMVKNSPLYGKIWF_DQIRRNHSLLA_LCMQMVI_YFSIKLYQLIYIYFTKWMNKSGSESKICKIINPVK_FLTTMI_VLTVSSE_TP_HRLKLKRPYHLLLSVTRVRKHNLKSYLLMNQNNPNQRLANPRALFTL_WPNQT_KT_SM_HTAFTRVLLLETRLISINTGE_QKKQSL_NKPGKKRCIRLKRMLN_HSIFLSTIIF_EILQSQMGVLYFWAIIFKKMEL_SSNTSRR_WLTLTPNYLTMNQ_NCTMMGLK_SLFGMSYI_KKTKKIRLLLLLYFTKMGILFSYMKIYRFLYKRFMTAKC__K_ESVMTI_VIPRVG_LSRTTI_QVGPS_NYLKFLYINALHYSHLLRLVPPLCPLKTRLVCLQSKIIKMTVCHQVTHLT

**prophenoloxidase** (294) [122443] NYGQRNEKPYAVVRQA@RTAHHT@RR@@QPISTYGPIFVERLREQWPGHKQPFRRERQREDSAQESAAAAGTQQVSAAAL@CGLLAFPA@TPGDGQRGYRRAFGCS@ESAAXXXXXXXXXXXXXXXXXXXXRNDTKNVPIQNFAETFPAKFLNSQVFSQARETSAVIPESIARTPVIIPRDYTATDLEEEHRLAYFREDIGINLHHWHWHLVNPFTASDIRIVAKDRRGELFFYMHQQIIARYNCERLNNSLKRVQKFNNWRDPIPEAYFPKLDSLTSSRGWPPRQSNMTWQDLNRPVDGLNVTVSDMERWRRNLEEAISTGMVKMRDGSTRALDIDTLGNMVEASILSPNIDYYGSVHNNGHSFSAYMHDPQHRYLESFGVIGDEATTMRDPFFYRWHAWVDDLFQRHKESPFVRPYTQSELLNPGVQVTGVRVEGGGQRNVLNTFWMQSDVDLSRGLDFSNRGPVYARFTHLNHRDFTYFIDVNNTGSSRRATVRIFLAPKLDERQLPWALSDQRKMFIEMDKFVVPLNSGQNTITRKSTQSTVTIPFEQTFRDLSRQGEDPRAPGLAAFNYCGCGWPQHMLVPKGTEAGAQYQLFVMLSNYELDKVDDPNAGAGGNTTCMEALSYCGLRDRLYPDKRAMGYPFDRPSSAAASIENFILPNMALQDVTIKLQNSTEPNPRNPRQ@TTTWLLYSLTED@G@IG@NQI@LDHYNLKE@NLPXXXXXXXXXXXXXXXXXXXXXXXXXXXXXXXXXXXXXXXXXXXXXNYYFILAFKKRKVVNLSKES@VLYILYL@G@K@ARIMIRCY@LPK@IKDV@NIYFIFHCCKETHCTCQID@KPLCENFKKLIYYHIGIFISNS@NQGYRLIQN@Y@IHTSLLSQTTKTERSALDHSSVHHGRAHSPTIIRLKSFIRSLELFFDLKV@NQLNKVIVETVRAHIFM@MF@T@TTMKYVSLHTVYNELIILACHFQAVFC@SN@NTLWHVSANNTTPFSA@Q@TA@SKK@EVPYTIQNYDKTRAICEQFALFTNRTCQMPNANHKLTRLM@@QA@MV@LNGEQL@CS@RLERKGVAPWHKLLRGVVTNNNYILGS@L@IPSKLI@NPSN

**prophenoloxidase** (339) [84833] TSNMSGNKRDLILFFDRPTEPCFMQKGDDKTVLQLPDTFYTEKYRPLSDRLSERFGSDAGRTIPIRNIALPDLSLPLQLPFHDQFSLWVPKHRKMAAKLIDIFLGMRDVEDLQSICSYCQLRINPYMFNYCLSVAILHRPDTKGVDLPTFAETFPDKFMDPKVFRRAREVSNVVPNGTRMPINIPVNYTASNQDPEQRCAYFREDVGVNLHHWHWHLVYPFDSTDRTIVDKDRRGELFYYMHQQLVARYNAERLCNNLGQVKRFNDFRAPIEEGYFPKMDSLVASRTWPPRFANTSIKDLDRPVDQIKVDVSQMEIWRDRFIQAVESGSILLPNGRQQALDETTGIDVLGNLMESSIISRNRNYYGDFHNMGHVFISYAHDPDHRHLELFGVMGDSTTAMRDPVFYRWHSYIDDLFNLHKKKLTPYGDRLVFPGIRVSSVGVEGRAGANQFGTFWQQSTLDLARGLDFTPRGSVXXXXXXXXXXXXXXXXXXXXXXAGRDGHGAHLPGAGVRRERAAVPLRGAQADDDRAGQVHHAAEAGRQHGAPAQPRLVGDHPVRAHVPQPERAPRRPGHGAGGRVRLLRLRLAAPHAAAEGHPRRVPVRALRHGHQLERGQDRARPSGVVQRRGVVLRHPRPQVPRPPRDGLPVRPPGDP@RHDVERLPHTQHGDQTVPHPVL@RHQAKGPAVDAYKENKKLNHTFKEIR@KFFIRNK@RGRGTAWEAFASKTYDAVLCLLILCVPCSFY

**Serine carboxypeptidase** (209) [66343] LLKHFPVSISKLPYLND_HRPYCITLLRNSVLGSGFKQYSYL_KVH_K__SINSFIYQ_CIPI_KVN_FCRA_TLRHYQQFTNLITLCHKFPKMKAAINFVLLLISYGAGNVYPDSYPRVTMKASDNSSAGEPLMLTPFLKNGSVSQAQRLSRVSITDELGFVSHAGFFTVDEKYNSNMYFWYFPPICGDNTAPVLLWVEGGPGASGLYGLFSGVGPLKTTKHGFAKRKYNWAEKYHVIFVDSPVGSGYSFTDSDDGYCTDEDCVAKGLYSFMRQFYQMFPDLKDNDFYLTGESYAGKYIPSFGMEIHQRNLDEREACKINFKGMVMGNAYCDPITQTVYGIYLYQHGLIDYNQLQMFEALQNEVIEAIKREDWTVANERAEKFFGNGSYFQEFTGFENTFNYLETKERDYFTRLLELLNVNANRRSVHIGNLQLSDGLKAIEKLAGDVLLTVAPKVAELLNYYPTMFFSGQLDLQVSYPGTENFLRHLNFSSAEEYKSAPRHIWRVEGDIAGYYKTAGNLTEVLVRNAGHTASQDKPKWVLDLLNRFIDGDLK_IVFITATVLNVNC_LSYKKLKLLLHIKCFKDK

**serine protease** (88) [131960] I_KIFLFVSKHLIARWSSACG_LTHMYPTESVECEPRVGSLDEDKASCIQ__IIILE_TKG__VNVNITLIKSISVITMISYKVVVIYCLHVASLSSVFAQRCNGGAQCVTIQQCSGLYKQLQDGNTPELTRLLRSLHCGFQDEDPKICCPPEFRGIKNQGSTVPTQSTRVGAPSSLLPDFTKCGIQFRDRIVGGTQADLDEHPWMALIRYDKPNGWGFYCGGVMISHRYILTAAHCVKGQGLPPTWRLSQVRLGEYNIKTAQDCTSEYCSDAPVDVQVEETIVHENYDAEDNNQQNDIALLRLSQDIPYSDYIQPICLPTTNALKNSNYDGYRAEVAGWGKTETRSESDVKLKVQVPVVSNTECSNVFGNVGRRISNKQLCAGGEPGKDSCRGDSGGSLMVQTPNENWVAIGVVSYGPSPCGTPGWPGVYTRVGAFIDWITSHMRP_LSFVRD_IFFKNFL_IILRQLYNEE_NHNLTNYYFTWLYFCFLTKS_FNTLHSRYSKYGKIRLC_KNDGEIDGPTSF_PKHCKCEFYALCPLLKTYKI_IHEKRLMTVAKWIQYGV_LQPKSWHNRIICLGIVQNTKSEEKA_FDDH_V_VIEIRCK_QKNTDWTIVRA_YELVIAFT_YKRC_NLYIGRYFKILSEIRASYFWLINVIYLDIDKQ_CVNLH_EILIKXXXXXXXXXXXXXXXXXLHT_TM__EQQHIIKKRSLTLNKSF_TGNLISWVNMLLLICVYLSIFFYKN_FRNFLLQ_KIVKYGKYK_NNNKNFLIY_KIKN_IEITIYFPHTT_LVYIGYLIIY_GRYETT_DFGYLNEIKIFIYLAKTNFQECIFASGMFTIDMLFSISKSY_AEKIS_KYYCD__YFY_AALKSCLLNNMAYFTIPTSFHKHNKKILK_RCYKTTVFCTYS_VLIFL_LYKNK_IFLHKLRSSYYFTRHIILLGI_IFNNFFRWISISIALFHFRLIFLYFLK_MFFNKTIRITICFWTILDKFFKLFTKFNVHLCYTF_ESFLKFICPCWKPFIE_FNPTLKQAHGFILT_ESCQGILQLIWLSTASSC_QRQKVITLQFTELFKPLK_IPQLCFRCQPG_HVAYEGPVLFKFYTYASRWDGPFKFSMSSVLL_LYR_ILLNLTLWLYIFQGITKQKAKQNLQTF_LQ_HNNYIQATTL_ASIAQQTT_

**serine protease** (93) [51666] GNITLEYTVSFIMFSSVFLLLFYGSITSLKADASGQTSKRCILPPFPSNGSYEVLGQPSAAPGEAFDSVHLKVHCNPGVELTKNNDLFCVQGAWSDEMPKCIRTCRLNTHPSVSYRCSTPGSNEGRRDCEQNEPVGTVVEPVCKPGFYLPVDLRDMHCLDGSWDYVAFCVPDCGVSNPESLILDGPSSLRGKVPWQAGIYRKERNERYHQICSGSLVTEKFVISAAHCFHTESTRVLPPSNFLITLGKIYRDLNESRDTGAQISDVEDIKVPPRFTGSTTNYQADIALVKLVSPIRYNRFTRPICLDFNINFERQQLQDGNLGMFAGWGISPVTGVSSQILNIRNLPYVDITRCIAETPINSRELITSDKMCAGRKATAGACRGDAGGGLAFPEVSRGVTRFYLRGVLSTAPASDDLCNTDNYVFFTEVSKYEQYIKSLLLEL_RR__KNFFLLKISFIS

**serine protease** (238) [50620] DE__QDSLSGNRRKSTTSSVHFIKKKLTNHFTVYQIFVFKT_N_RDTHKRCNIPTYYCTRHNINSNRRRDE__QK__VISNLLCDDVMPISSSI_ACYLPVKRNTSTMFGDRRLTVILAVVVLAGCARARPNGDSIEDNTITSNVSGAPQPQPQESTPTPAPVPVNEQFPHAVLFGGTCGGSIISPNWVITAGHCSIFTSGRYVLAGTNNSDDGSGTYVHVKRLVIHPRFSVGPYWVDADDFNIQQVGARYDYMLAELQEPLPLDGKKMAAIKLDDQPSVPVGLEVGYAGYGAENHGETMRSQMHGMDLMIMPDQTCEKLEQYDKLDMLCTQGRPPKLDSACNGDSGSGLVGGGRLLGVASWVENDSHECRKGALVVFSRLSLVRDWIRTVTGV_HFSYTLSLVYTKPTLAIRSQ_DWQAALACYWPRINSFSS_ASADCHNRAPVYFSSQ_LAPALLASCKLYL

**serine protease** (421) [149433] PSRVPVGNMKFVYPDARRDESVVDDYHGTKIPDPYRWLEDPDSPETKQFIDAQNNITRPYLDSCPIKADVHSRLTELWDYPKYSCPFRRGDRYFFYKNTGLQNQNVLYVQDSLEGEPRVFLDPNTLSEDGTVAISGSRFTEDGATFAYGLSASGSDWITIHLKDVETGRDYPEVLEKVKFATMSWTKDHKGLFYSRYPEQQGKTDGSETEVNRDQKLCYHRLNTPQAEDVVVVEFPEEPLWRIGAEVSDCGRYLIVSPVKDCRDNLLYYADLSKQPEVAGKLPLTQIVHKFEADYEYVTNEGSVAIFRTNKNASNYRLIQIDFENPDEENWQTLIPEHETDVLDWATAVDGDKLVVHYIRDVKSVLQLHSLKSGELLQTFELDVGSVVGFSGKKQQTEIFYQFMSFLTPGVIYHVDFKKQPAKPTVFREVTVKGFDASNYEAKQIFYSSKDGTKVPMFIVSKKELPRDGSNPVLLYGYGGFNINVQPAFSVTRLVFMQHFDGVLAIPNIRGGGEYGERWHNAGRLLNKQNVFDDFHAAAEYLVRERYTRPALLTAQGGSNGGLLVAACANQRPDLYGAAIVQVGVLDMLRFHKFTIGHAWVSDYGSSDDEVQFHNLLKYSPLHNIHVPTDEKSEYPATLVLTADHDDRVVPLHSLKFIAALQHAAAAPAARQRRPLLARVDTKAGHGGGKPTAKTIEEYTDVLCFMSQALGLKFHK_QQPRAAPATRRV_QTR_RKIVL_CTYINVNHNHRLPMIASGGGSPSRETGVIFFTH_LFF__IFVFIFESWFPSTNLEYNLLYSKNEMLFYNIFNLYYPYKC_QRIVNPMGLAPSTMIKHISNIQ_NKINNFRSALSRLYSNNLNYANFFXXXXXXXXXXXXLVSDFCKHEEIISLASISNS_FKGTKCILSN_Y_NV_SLFVLFEEQFKYLFNLPQTLVYVKKYIIRKFSAFYQVRFCFHEIVKNTNKKI_IEK_Q_WYLSVITDVSSAFSYS_KK_K_RRYVCIT_CSRKSIFLVFTFN_IIY_VXXXXXXXXXXXXXXXXXXXXXXXXXXXXXXXXXXXXXXXXX_STFS__RDCTRRVPRAIR_IESDLNHSCHQRAKKLSPIRCALVFRLFIRIIINHGLVIFEQINNILIVSL_FQLYYSLYFTKSKYTYLFVQSA_NITYRRYTKITHIQSLGPERLFTNYINRKNIHLKIEAIML_NRRSARYLITNRTNKKTLDAVASLGGRSLNVTTLNCQHVYKSVCAR_KKTAIVSTIGYLEIGLVIIFCGYTILSDSQKHVRIYECIVFYVMTGRWTNTYRRTIKLELILKKDLQSSFILR

**serine protease** (427) [85396] PCPNVFQ_VCFVSPSS_SASHTPT_TLLYIYFCFHES_VF_YKKMFRAACWCLVLVLAVPSHEQSTPLSPCPNVFQYEPTGSEPGRWYGVVNLSTDSILHSLWLNVVLDSKADILGNWVGDVSTTDNVDFKIENTKMKIQPGPATAVRFFVQYNPLNKAPKLLAIRLNGREICNANLPSPVVDRPDSDYVRPETTTSRSISRPDGTKPRPGEIRPAEKRPSENKPVDTRPIFRPEDGQETKPVKGTPSVGGPVYVPTPSSDQGSANPYSIGGGQYPSQNAVVSSGGSGSRTDTTYVLTTTAASRRRHTNRPSVDDTDYSDSKPDPDDYFSGPRPVIPVVKPDRNNNNNRGQSECGKVVRNNPNPLVVNGKPTLEGQWPWQIALYQTRVVDNKYICGGTLISNRHVVTAAHCVTLKSSSRVVNKNTLTVYLGKHNLRTSIEGVQIRFVGDITVHPQYNASSFSKDVSVLELLETVQYTDWVRPVCLWPEEQTDLSNVIGKTGSVVGWGFDETGVATEELSLVDMPVVDQETCIRSYSEFFTRFTSEYTYCAGFRDGTSVCNGDSGGGMVFNMHNTWYLRGLVSLSVAKQADRCXXXXXXXXXXXXXXXXXXXXXXXXXXXXXXXXXTIGI_HFLDRDKRVF_INSVDFYEYVYIVD_KN_VQYIV_CLCIYS_GRESPITAYVQ_RPLSYIP_IILIILSERIERWEPLSIETSEHSRPSAPKIKLVII_FLQYVHYIVYVLRTIKTSPYSHKLSLY_RYL_TV_YMVNMDLQ

**serine protease** (813) [76084] ASIQ@NQVSSPRLRSYQ@LAETPRSWWSGSRDSQHSSQHGAPSLAPLLGACLSFATPAPNTAPDACHPRKFRYNNIPTPASMSGDAEYMTTNYNMRNNVKLSFWGQRTKLEKRLILVAGVTGVLAIAFLSAFIASLVLRPVTTISDMPQTSELRLSSPAPPAIISKGDINLKICNSPGCIHTASKLLLNMDDDVDPCDNFYDFACGSFLKNTRIPDDKTSINTFSIITDQLQEQIRALLDEPITPEEPKPFVLAKKLYQACMNRTAIEARGVQPLLDMLRRLGGWPVLDGDHWDEKSFAWEQSVYKFRAAGYSVDYFIDFSISVDVKNSTKRIIDLDQASLGLSREYLNRGINDKLVQAYYEYMVDIAVLLGAERNRAKADLYESLQFEIKLANISLPLEKRRNATSLYNPMTISQLQEKFPKIPWLDYINQLLAPHVKVTPDEVTIVNVPKYIEDLEMLLEKTPKRVLANYVMWRVAGASVSYLTDDLRRRQLAYVTALSGKTERESRWKECADTTSVSMSIAVGALYIRKYFDEDSKANALEMVNDIRQQFRNTLTKVDWMDEATRSAALEKADAMSAHIAYPSEMLDNEKLTEFYNGLEMSSDQLMESVLNLTLFGTEYLFKKLRDPVNKTDWVTHGRPAVVNAYYSSIENSIQFPAGILQGAFFSAERPAYMNYGAIGFVIGHEITHGFDDQGRQFDKNGNLVDWWQEITKQKYLEKAKCIIDQYSNYTVKEVGMKLNGVNTQGENIADNGGIKEAYYAYEAWTNRHGEEYRLPGLEKYSPRQLFWLSAANTWCAVYRNEAIKLRITTGFHSPGRFRVIGPLSNMEEFANDFKCPAGAEMNPTKKCKVW@Y@VPATTQAHVTGWYIHYHDVKGPAESQNELTYYLK@NNYL@KVT@QQTLLLV@WSVTEEAKYRNASVVPLQYRCRSRCLRGTFYCTVTEVRHLVGRLVRECVV@MLTASLGS@LLVCK@@@IIIVSNSESLKM@YLHMARSLYRYYLNFFLLD@AQIILT@YIRVKQEPVFFLLSKSLFLIKIV@LFIFYYLQSDLYVMLDKNFNGN@@NYFFIINCGQHN@CTYLIKILFIS@L@@@VYMYLMILELLELIVLLPTGFQDPIGLMIFMIDFMIY@FYILLLYKRRYFSIVLHDYRSS@IRSLVYNFEQR@SLNGRRDS@SCSDSVSNIAVYCIESHSMEFETSSDILVLLVYLSN@VYIFGYDRIVVNH@NTNIINFYV >Zf_C676 15231

**serine protease** (1085) [41745] NGKCVAERERQCVI_SHHVIITFYNIQIHVDNNMYNWYLL_VYINQ_SKAKVVYIGDYSTMLSFVVFKYLALVVSLYGANGKCVAEQDDDDTFFNYHERIGIPTALQIKEREDKALSAPGLEDRISGGDVVSQDLVPYLAGIQITIRGKSSPSLCGSSLLSPDRLVTAAHCWYDGTSQATNFTMVFASQYLFYGGVRISASAVVVHPQWNPKTFANDIAMIYISEKVEYATNVQPIGLPTPDLRTETLVGTQVLASGYGKTSDLEAYVSVETTVRQVMLTVISEQECKQYYGVLKVRSTNLCTSGKGGVGICGGDSGGPLVAIRDGKHVLVGISSFVAKNACHAGHPAAFTRVSGYLDFIQAEQECKQYYGVLKVRSTN

**serine protease inhibitor** (83) [57781] VSQSSAILCAYCFCFQ__TDV_RIKERNI_LIRVIIAVLRTQ_FFYQ_YSAPKVTMVHLIYATFVIFTITILKIGNCQLSIKQQQHGQIHFSNDAPSSPAWKGKNPVTPSQVKPVYPESEMDKPRLEENLHNTNPPSIVDDSITFIQESDYDDVMQSFPIPNNHHQADNVSLAITKFSLNLMKSIDQSQGGNVVVSPYSIATLLALFVQGTTGDTRLQISRALRMSPEYSESSFKTIIEDIGTRNSPQNILCVANNVYVSNTFNINREFQSKAVNNFASEVTPMSFADPTKAVDDINYWVALKTRNMIKKLLSIQNIDDETQLVLANAIYFKGAWQKKFKKQSTSLQPFYLASGTTKMVSCMHSRHQFRAGIDRLNKAKVLVMPFAEREYSLVVLLPDKKSSVDTILATLSSEQLTAYQTLEPIDVVLTIPKFAIRADTDLKSVLKKMGITSMFSPNSELIGLGTSRGMAPQVSTAVHSALLSIDEEGAEAAAATAFAAVALSYGGPDFEFKVDRPFVAVLWD

**serine protease inhibitor** (110) [61034] FTSPVTTVYKPLPTYI_KQISCRQTGRCTCPN_IFMSAIKYKINIQVFCSSISLLIIIIMNSLSCFVLILGCLHSLECLEQNRFFEKYRMSFFDTDLLYYTAVDRKGNVAISSSGLKSILAMMLEGATGGTAEEISSALRLSPDKDVKQQLIYYFTYLQSEPARNILQSTNIFFVSNKTEVNSKYKRLLQDVYSTEIKEVDFSKPLVAANTINARVSNATGGVFKNFIEPSQINPSASSVIANTLYFKSSWMYAFDAKETRNACFHVDGACRTVPMMELEKQLNYTYVESLRTHAIELPYEDSRYSMILLVPKDRNGVNAVIRDLSYMSLPLITKEFEPTEVYLTMPRFKIDFSEDMAEVLKKMHIYTLFSPAANLTGIFEDEYIFLNDIYHKVHMSVDEEGTVAAAVSGGMIAPLIGGRVQLAVDRPFLFFIRDNLEGVVLFEGKIEEPTASIVQNLTNTQETAEILKEIVTAAPDVPQNANNVKGIKPLNVDTAIPDKAQKYDDNIPVSNIPRFAKLRKYPISLSNGRQFFTM_NKII_

**serine protease inhibitor** (118) [14612] EEQFYRANDEFTGKMFMEMVKNKSDTSFVQSAYSVLPLLGLLGLATVGESKDEILRTTGLSDDDAIKAVFPVVESRLRSIKGVELKTATKVYIPIGYELDDNFAATARNTFGSEVKNVDFTKN

**serine protease inhibitor** (135) [61378] GVGQLLSGTGLGHALSGAGVGQFLSGAGVGQLLSGNGAGHALTAEKLARKELVTKFGLSLYPFSAQFYKVTARYHNYHFVHSDYSVWLAMAAMAEGATGDDQKELLQAINMPDSESVRKEYYALAKTFEVPGDDVVLRRKRYLFVDEAATLNSSWASEVRTLGLLEALSFPLCADPQRALALVGLPALPANLPALEGHSLFADSLDFETVLTTAFADAYIERDAPFYDNGGRLLGSVDLMRFEKRVKLAHVPLLNVKAIEIPVGLNGRYRLLMTLNLGNTTIRDSVENLQPTIIGEILDNIQESAIPLQVGIPVFSMSSVFPDVRALLERTRSGVRRLWSEPASSARVSAPPALPSAYVHRVSLRLGAAPAAGAARPLPGLQVVSELATGLAAALGRDFIANRPFMYALVDAETRIHSFSGVFSLPDKPK_FICDTFASKLIVPK_ISTPPVRFLYSGSLILYLYYLFFTSSKYQCTTNLNFKK_HCLQNCLKRSNCIYKMCNGKVREILKVAAPLPECVFLLVVQQEVVVVVVTLFLRL_RPHSESTYYYLTKYRILKQ

**serine protease inhibitor** (143) [13673] PVPQEYEICYRDVTQEVSRATLDFNLQLFKVSANYYNNQFVLAPYSIWMTLAALAEGASGFARARLFNVLNLPSQQCVRQNYYKAMKHHERPCSDVKLFRNRLLGYDECLYLNET

**serine protease inhibitor** (157) [53461] LELVVIGIHEPS_NPTRLSCRSSIKSHT_VLANQLNDIHCAGNT_ETSIRGSYKFSRRHYCLKHVRDDVRAMFKALLNLAIASLAVAAPSKSPKESQFAPINKFTFKLLDNTFVFQENFGRKNFAVSPLSVWNAFALLTEGSAGDTFQELMTSLNLPKDLKATQALHRAASDVVKYNSEDVVLNGQSAIFADGSLAINTEFCEAATAYNTDIYSVHTKNTTALANDINEYMCLATQGKVTDAVEPDTLEDLVMLLIDALYFKANWTHSFDESDTTVLPFYNYHGKIIGTVNMMYREAPLNLLYSTDLKAEVLEMPYGDAKDFSMIVLLPFDGVPMKNFLHTLSSAPLGWLKELKAANSSEKVECLLPRFKISCQVDLSTPLKYSGIHSIFDSEKAQLPGVSATPLFVSKALQNVKVEVNEMGTEAAAATVIGFETRFGAPVFKANKEFVFLVIDRRSDVILFAGVYDTPSLV_TF_TP_YVC_S_SLSS

**serine protease inhibitor** (221) [23369] LDDNFAATARNTFGSEVKNVDFTKSGETAKEINDWVEDQTNHRIKDLVDPNTLDQDTRAVLVNALYFKGSWKQKFDSRYTXXXXXXXXXXXXKLPFMFQNNDYEYYESQDLDAKFLKLPYEGEEASFVIVLPNQIDGLEALEKNLTPEAFNNAMSNLRKVEVNVHIPKFKIETTTDLKAALSNIGINALFDPSKARIDKLLKEST

**serine protease inhibitor** (241) [57096] F_IARCQTDVVHP_AIPSFN_SLYIFNRFVNNISSASDDFTIPIK_P__NCKHKADVIDELRTKNINTCVLKFETTLWV_TLFIYCVSCLLLLTGKMLSSN_MKPSCPQTYTMDSRRKLAISHWNCFTIQRNRRMGIT_SCHRSLFGPYWQ_QLKVLLGTHWLR_TMLCAWNSTKLSQDLASKMSRGGFKYTQTLLNWQN_MRYLLMKKSYR_KISEA_PKIIMIQSW_R_TIVKLVR_QSIS_MMQXXXXXXXXXXXXXXXXXXXXXXXXXXXXXXXXXXXXXXXXXXXXXXXXXXXXXXXXXXXXXXXXXXXXXXXXXXXXXXXXXXXXXXVSLQDMFRNMKTVPMDTLFSELRIAREDYSDEEVDLFLPRFKIQSNLVLTDMLKNNFGIVDLFEKFSARLPHMSRVPLYVTKIIHKAAIEVTEEGSTAAGVTVAEFANRIGITRFEANRPFSYMIVEKVTNTVVFGGIYATPSLY_TRMMPELKLRSD_FLSVMQVSKLFCT_IQKKRR

**serine protease inhibitor** (280) [26258] SIKGVELKTATKVYIPVGYELDDNFAASARNIFESEVKNVDFTKNVETAKEINDWVEDQTNHRIKDLVNPITLDQDTRAVLVNALYFKGSWKKKFDSRYTXXXXXXXXXXXXXXXXXXXXXXXXXXXXXXXAKFLKLPYEGEEASFVIVLPNQIDGLEALEKKLTPEAFNNAMSNLRKVEVNVHIPKFKIETTTDLRAALSNIGIKALFDPYKARLDKLLKESTNLYVSKAV

**serine protease inhibitor** (595) [72493] KLH_NPLPSLNDLSAQTAGSYFALYYLKM_IRNSFE__KKNLLPYKYWQVN_FFFCLETV_E__AYKTYYSRNICNCRAREPT_LIFQNF_NFIQVQKRTLIFYLG_VSTTNKIQIQWKKLRTNVALELACVNTPQLYKRIPFVNXXXXXXXXXXXXXXXXXXXXXXXXXXIFGIKIFRLGAQHYKNQFVMSSYGVYLGLAATAEGATGDARKEIFDVLGLPQEKCLRDKYYKYILSAEPRSSSFRKPDGYLRTRALVVDENFKINNTWAEDAQKHGLLNYVLPLPLRRNKEDAFDSLNSLFHNQIGNLDLDGNTLIDSIDYDLIWQVEFPESQIQHDAEFYDDSGHEIGRVDLMRFSKHAKIAYLEKYNAKVLVMPVDQDGWYNMMMLINIDSTPLEQTVASLDEGIIDLVVKNMKKSEIPIDIAIPRFSVESNRQDSLKLLLEQMGVKKLWSDPEGSELISSPPAVPSAFYQRVKFNVGSEGALRYFQEESLGNMSYLDGGVVDFIANRPFVFALVDSRTKVRLFCGGYSGSELRRGH_I_PIEILLNVXXXXXXXXXXXXXXXXXXXXXXXLTDRTEVAF__SHNFK_RFLKQNCCNHRR_LNKC_LAFN_KLN___VIIKTTQSRRRVQPCSFAVPDFS_IYLST

**serine protease inhibitor** (614) [79136] RVTCVTSFNRSPPLRFTIIFSRGLDSRLLCTVLCCRYLENKIKQMKYSQLKVILNVGNKL@N@NFT@NKYFLVELIPVHVGTYLELKVFLKVVFLVFNTGVKMTLSLCFFMLFVVPPLLSADAPLPTIDSNTLHNVFGYPDAPILAQSVAPLVLTDPAAQAVVLEVAQAIAVRPINFTVVDEQFVQSTLDYDKFDWVLTKRVAASSNENFLLSPLGLKLALAILTEAATGVTQAELANVLGFELNTLAVRQKFSTIINSLESQSSQYVLNLGSRIYLDDYVHPQQRFAAIAEEFYKTQLVGLDFSNTTVAANEINSWVANKTAGNIKMLVNPDDLGGIVAMVLTTIYFKGTWCHQFPPNNTRLAPFYISPKAQAPVPYMHVRNRFYYSESVEYDAKILRLPYMGNKFSMYLIVPNTLTGLPRVLNALNAANLRSAILSLKESIVDVTIPKFHFDYTSVLDSVLQELGIRQAFQDLASFPGIARXXXXXXXXXXXXXXXXXXXXXXXXXVKLTQLQKYL@RTNLARTRKLFWRCWLTSRSCFSFKTRRPDSFSSRDKWPIPFLLTELLNFHKNLNDMV@FCRSQRNFIFVHTYLLHS@KKASFYKVK@NISMF@MCRVPKFTWSTFGFL@YLMFSLCPIKINSILIIYLKEIK@HVYT@VPKIVSMELCIRDKII@TLTLYLCRRL@VGKE@NKMSLNA@KKKK

**serine protease inhibitor** (644) [33892] SRTPRRPLQLSTIGRKPKPRVTSKIRCQKTPSIRPS@PLY@MLYSSRAIGVFRLTLARQKRRTSA@AVTSXXXXXXXXXXXXXXXXXXXXXAKLVELPYKETGFRMVVVLPNAADGLPELLKKVEQHGLLSDVFQLEPAGVEIDVRMPKFDIESELDLTTLLPKMGISKLFSDAASGIVKGDSVVVSKAFHKAFARIDEEGATAGAFXXXXXXXXXXXXXXXXXXXXXXXXXXIHCCAVVFKLQITAADALPR@PSVPVHNYVPGQDTFYRHLHTLKYLFAGVGVYEYLSRIKN@LIKK

**serine protease inhibitor** (705) [15884] KVPLDDLCVTGNSVLVDTFDYKGLWSSAFPNVTIEHNYPFYNENDEKRGEVDLLRIKRIIKYAYLPKYSCTVVAIPVKENEPFSLFIVVPVEGTPMRTTIESFGKTFLEDAFDNFHESTKPVEVAIPRFHIESYHDN

**serine protease inhibitor** (1403) [46939] VIMKLLAVAVLVYACVAIGDPTPEDVCSRDVSKEVMPAVEDFNIRIFKATSQHYNNHFVLSLYSIWLTLSAMAEGATGAAQERLFDVLNLPKEQCLREKYYKVIRKHERACSDLKRFRKRLLAYDDGLCLNATWASQVSGEGLLEPVEVPIRTDPELAKRQLSDLAKTSLDYVSLSGDSLLLDTFDYAGLWSSAFPNSFIEKNAPFYSDSGNTVGSVDYIHIKRICKLAYLPQFGFKILEIPVDNKGHYSLLFFLTTDSLPVAHSVSLLTKTILDDILANLRQSLVPIRVALPRFKIASHHDDMQIVLEQIGLSGLFKDPAASELVSDPPAMPSAYIQHVSVNIDIEAEDYHEEVEEASDVETGWAKEAGRTFYVNRPFVFAFLNNRSKATFFSGAYSQPSASA_IFLQFFVVVIIVC

**serine protease inhibitor** (1406) [80377] RMFHALGLPEEACLRQKYYRRVKRHEKPCAGLKMSRSRLFGIDESLTLNTTWAEKVRSAGLLNTVILPLVNNPEAAVERLNALSKVPLDDLCVTGNSVLVDTFDYKGLWSSAFPSVTIEHNYPFYNENDEKRGEVDLLRIKRIIKYAYLPKYSCTVVGIPVKEKEPFSLFIIVPVDGTPLRTVIESLGKTILEDAIANFRESTKPVEVAIPRFQIETYHDNLQVVLERMGITGIFTDPTAIEYVSQPPAVIGEYVQRIEVNLDLTGEDYREEEEECADEENVPLQEIRNKYVFNKPFIFTLMNTNTRACIFAGAYSKPSP_FKSTYWNSSK_SSLLYDGFYLKFGNLFLG_STNVRTQ_KL_SKAMLCLLLTLFSCKLCCN_FMVGAMHISESALNI_VYKILWMELAVG_SPALFFIF_TVLFNLPWLFFLFRYYSILFIQTK_I_QNPN_LYFQMC_LYF_IS_QLI_DT__KQNSNN_NANVSLFVHLLRFYALMALSIIMKLHVMYFKARHERARHRV_TDLKGSAYY_IIRFSIFWIKFCHFPLFSIVHFYVFVKLLDDHAIEPKKNLAPVLPNRLVSI_MTLFD_YCNLVYLGLF_PLHLFNRLPKRRRFYI_LCVCFFRY_QTSNLLVVSRSLSHYLFKLFCAPNSSKSPSKVWRDRKRTNIFTEIS_YYSTIYGVILMITQF_VKIFKIKIAAIRDVN_LMLIGI_L

**serine protease inhibitor** (1567) [69495] RRPAVDHGVRCITHTGLSQPCSCWALWFSFRRAWSTAGRRCRKSTKXXXXXXXXXXXXXXXXXXXXXXXXXXXXXXXXXXXXXXXXXXXXXXXXXXXXXXXERPCSDVKLFRNRLLGYDECLYLNETWSIDVHNNGLLDTVSLPILSDPEFAVDRLSYYSKAPLEDLCLTSNSVLVDTLDYYGFWSSAFPKASIERDAPFYNEAGVSIGKVDYIHTKRFAKFGYSPQSRMRIVEIPVDLKGTYSLLIHLPDDYVPLKAVISELTPSLVEQNLENLRDSLVPIEIALPRIKLTSYHEDLPLFLDQIELTGLFKDPATTEYISDPPALPGAYVQRIAVNLGVTGEDYREEDEDCSDLETGLPEQFGNSFYVNKPFYFVLINTKSKIALFTGAYSRPDA_LNSLCDFKV_LFFFIFVFV_IINLLFHFFFIDCKTK_VCIVSTKICYLLRMFVIVQGQXXXXXXXXXXXXXXXXXXXXXXXXXXXXXXXXXXXXXXXXXXXXXXXXXXFIIFILQYDSDR_NLTLK_NDLIIIFAPWGNQN_REYNSAVIETRVYNKNYAAISEFLREFQHKLYV_TFLLNHTIYR_KLH_NPLRSLNDRSVQTAGSYFALYYV

**serine protease inhibitor** (83) [16248] ASASCQSVDDLCSRDITKEFAPAAVDFDTRLYKAAARHYNNHFVISQYSTWLTLSGIAEGASEATQEKLFDALHLPKEACLRAKFYKSVRKHEKPCTDLKIFRRRFLAYDESLTLNSTWLERVTNAGLITAVSAPLKSNPDLA

**split ends-like** (72) [56672] NVQKQQDEIKRLQAQLLLNQQQHDQQQQNNKQYEHNYNQERNYYNQKQSQPTSARPQYSQERSRNPTNFVTTAPSHYQSTSRTVEIKPTTQKYVSSTQNNNLAKKEERKQPNVELPDEVPDDLRQQLLSSGILDNADISILDYDKVGETPLDSLPPDQLANFFSAGGGQHLASSENRPVVVKPNGDXXXXXXXXXXXXXXAIPPSSQKQAVDMKVVHFDPNTAKGQNIASEYVKKDATQLEPVSLNDKKYNRYLPLKVSGNQFPVPDILKGRKITSVVVLAPVETEALNGDHTRAERATSSSLKGIKFIAGDSLQDLLKRPTKENFERWLETEKKTAADLQSVVLLVLGNEDQSEDREIFMYDITSGSVNKLSGELSNAFVDAAENNSFSKDLENVAIKGDGVPENFNRTDEENAEASENVPFFLDLSALNLDQENNNHVSISSGYSKTKVGRSIRRH_RSQNVARRLFL_IV_FYC__IKVT_CM_STVIKEMKMEML_NRNEIDFSNV

**sulfhydryl oxidase** (122) [89296] KNRVCTVSPE_H_LDKLTVELVDKQEV_SLHSAL_IERNSK_KLKIRRECCKRKLDIIILF_GNIITECMSVCLSKDEKCTVIRLLCFSTVILKTPTFDT_KMNYLLIVISIVFSFVAVNCAVVEDNGDVEEQGLYSKSDNVEILTRGNFDRKIYGKKHPWMVQFYNTYCGHCRSFAPKFKTAATDIIAWKNVIKLAVLDCSEEENNEICREFEVMAYPSLRYFHENYQKGRRNLGDFLIAPETADKFKALILFKMQNEQSLGRLTFAPPLKIESYATYTDVIRDVPKDTLYTFLIFENENSTTGCEIALDLNEFKNIRVKRAYGTSELADVAGIMRFPGLLAIGPNLEATQLTPINPVKTNLINAIKSFLQSRNYVLPTVGAAKVTTILANNPSAPNLINSDSDVVYYSDLEKTIKTSLHTEITRRKLLTGEPLQALINYLDVLISSFPFKGGNLKEYITDLKNTLTTRTEWDGSDIYELVTRLESTHSPVYISNLEYVGCKGSQSKYRGYTCGLWELYHTLLVNAADSPSLDGPKILRALHGYVKHFFGCTDCANHFQAMAVKNKLFDVKDNDKAVLWLWISHNEVNLRLAGDVTEDPEHPKIQYPSVSKCPACRLPRGSWNLQAVFEYLQSVYGSNNIQDTRHTRSAVAVPSFSNLDIGMLSLXXXXXXXXTCKPNNARQWRSRYPENRCLRTKPSHM_LDYKLLIPRIL__YYFLIIPKILLCDLESHSFVIIAKSIIYETFIVIICLFNILLTTLAVC_Y_LPIMEFRCRSKKNCKIN

**transferrin** (371) [40461] SRRLPRVDAVSTMTLKYVILLIALAGSGVNSKTSYKICVPSQHLQACQAMLEVPTKSKAILECVPARDRMECLVFVQQRQADIVPVDPEDMYVASKIPNQDFVVFQEYRTKDEPDAEFRYEAVIVIHKDLPVNSLDQLKGLKSCHTGVNRNVGYKIPLTMLMKLPIFPKMNDHSISPKENELRALSSFFSQSCIVGKWSPDLKTNSAWKSQYSQLCSLCEHPDKCDYPDDFSGYTGALKCLAHNGGQVAFTKVIFVRKFFGLPVGTIPASPSTENPDDFAYLCVDGSKVPVREKACSWAARPWQGLMGHNDVLAKLTPLKEKIKQLAEAGSTTQPEWFTKVLGLSDKIYHVADNVPI

**unknown** (194) [28878] GSSKPSTGLGSRVSLPATNNQTNTLTMMKAVILVLAVAALANAGTLRERRGLEGYYPQYQQSQYQPSQYGGGYGQDSDSVLSSLVPSGRFLPAYGQQFPQFRQQPSTFSSDVFDVNQYQRAWGTNTKSQGIYVPYSQQ_VIPFANELSTNGRLHRLPVQSKTFGNLCFCDYCYVDRHRPAPRCLLPARRPPASTL_LSVKNKTLLVLQ__KSANYYYGYVFVLNSIYHHVPILIHATEILLNALRIIVLLIAKIK_YYN_KK

**yellow-c** (264) [47161] RKL@FVALTACNRSLSCHSRGSAGLPFDPSMNRSK@ESFVSSSS@HW@LHAKRQHQI@GSNGRRYLIHGIPHKTEKMPYKTECMSQRIILYWDLQDGKTSYSLQCRDGRXXXXXXXXXXXXXXXXXXXXXXXDNLIADSAKELPTNSSIVSVFRVYVDACDRLWVMDTGIADRRGARNQIAGPSLLIFDLNTDKLIHRYFLKTTDIVEKSFFANVVVDVDSNTCDDAFAYVSDIKGYSLVVYSLKQDDSWRXXXXXXXXXXXXXGLEFQWTDGVFGLALSDPREDGFRTMYFHAMSSTKEFTVSTEILRNYTHIDQHKAVGEFKLLGDRGEKTQSSSSFYDPRTKVVFYTQVNRDGAGCWNTNKPFTVQNNPLLFTDPELLQCTIDLKVDDQGNLWLLSDRLQKLFFQSMDSSEVNYRIFSV

**yellow-d** (181) [64629] LVFRDKHTRSNIS_HTNQNYLKKNNLPFLPFIFK_LCFFFINLVCSRKSTILYPKLLNVFKLEMSHGMERFFLLSYCLACCWPGLQSAKSNLRVLRQWTELEFVFPSDSDRQKALESRAYVPGNSVPIDVDVQHRQNGQNSRIFVTIPRFDVGRPITLGTLDEQGRIVAYPDYSWHDNQGMNCDGLTSVFRVAVDECARLWVLDAGKIGDVQRCPPQLLAFDLESDKLLYRHKFNKTTYSDSSLFITPVVDVRGRAPADCSDTFVYSADVSGFALIVTDVARDRSWKISHRLFFPFPSRGTFTIDDESFDLMDGVLGMALSPWRNDDRYLYFHALASTTENVVRTKIIRNDSFIANPNAQANSIRVFPDERPIQSAAEAMDRNGIMYFGLMEPPSIWCWNSATDFTQSNFHRLAIDRETLQFASGIKVVTNVKGEQELWVLTSSFQRVMTGTLGSDRVNFRIHAENIPNLLEQSPCRNFPKDRLTGHHANLIAPTDFIQFSSRYGLESYL_KFAPRFT_CITV__MISGYTYLLYSLLIIAPIFKYMIGIFKKIF_KLKKKKKK

**Supplementary methods**

*LC-MS/MS*

One µl fresh defence droplet was diluted with 50 µl denaturation buffer consisting of 8 M urea, 2 mM DTT, 50 mM Tris-HCl, pH 8.1 for reduction and denaturation for 30 min at 56^°^C. Subsequently cysteines were alkylated by addition of iodoacetamide to a final concentration of 8 mM for 45 min in the dark at room temperature. The iodoacetamide was quenched by addition of DTT to a final concentration of 5 mM. The sample was diluted with 150 µl 50 mM Tris-HCl, pH 8.1, and 0.5 µg trypsin (Promega, sequencing grade, V5111) was added. The sample was incubated at 37°C overnight. Peptides from an aliquot of the digested sample were extracted, concentrated and desalted by reversed phase C18 STAGE purification^1^ and dried in a speed-vac. The proteolytic peptides were re-dissolved in 5 µl 0.1% trifluoroacetic acid and analysed by nano-reverse-phase LC-MS/MS. Chromatographic separation was performed by a Proxeon EASY-nLC system (Proxeon Biosystems) fitted with a 2-columns system consisting of a 2 cm trap-column ReproSil-Pur 120 AQ-C18, 5 μm (Dr Maisch GmbH) packed in 100 μm fused silica fritted with a kasil plug and connected to a 10 cm analytical column of ReproSil-Pur 120 AQ-C18, 3 μm (Dr Maisch GmbH) packed in a fused silica nanoelectrospray needle. The peptides were separated with a linear gradient (0–30% A in 60 min, and 30–100% B in 5 min at a flow rate of 250 nl/ min). Solvent A was composed of 0.1 % formic acid in water and solvent B was composed of 95% acetonitrile, 0.1% formic acid and 5% water. Mass spectra were acquired by electrospray ionization mass spectrometry in the positive ion mode with a Orbitrap Velos Pro mass spectrometer (Thermo Scientific) equipped with a nanoelectrospray ion source and Active Background Ion Reduction Device (ABIRD, ESI Source Solutions, Woburn MA). The electrospray voltage was kept at 2.3 kV with an ion transfer temperature of 270°C. Data-dependent acquisition was used for automated switching between MS mode in the orbitrap and MS/MS mode in the LTQ. 1,000,000 charges were injected in the orbitrap in which a parent ion scan from *m/z* 300–1,650 was performed with a target peak resolution of 60,000 at *m/z* 400. The ten most abundant ions with charge states > 1 intensity and above 10,000 counts were selected with an isolation width of 2.0 *m/z* units for MS/MS with collision-induced dissociation in the LTQ. Charges of 10,000 were accumulated in the ion trap for MS/MS, the normalized collision energy was set to 35% with activation q=0.25 and activation time 5 ms. *M/z* values ±10 ppm of precursor ions that were selected for MS/MS were subjected to a dynamic exclusion list for 30 s. Data of the Orbitrap Velos Pro were processed with Proteome Discoverer v. 1.4 (Thermo Scientific) and searched by MASCOT v. 2.3.02 (Matrix Science) against a *Z. filipendulae* transcriptome database^2^ in six reading frames. Search parameters were as follows: Trypsin cleavage specificity, allowing one missed cleavage site. Variable modifications: oxidation of methionine and deamidation of Asn and Gln. Fixed modification: carbamidomethyl on Cys. The peptide MS and MS/MS tolerances were set to 10 ppm and 0.8 Da, respectively. The significance threshold of the expected value of the peptide scores were adjusted by Proteome Discoverer to match a false discovery rate of 1% as assessed by decoy database searching.

*MALDI-TOF-MS/MS*

One µl fresh defence droplet was transferred into 100 µl water and vortexed vigorously for 5 sec. Immediately after dilution, 1 µl of the resulting solution was mixed directly on the Matrix-assisted laser desorption/ionization (MALDI) target with 1 µl MALDI matrix solution (10 mg/ml alpha-hydroxycyano cinnamic acid in 60% acetonitrile, 40% water, 0.1% TFA) and allowed to dry. Mass spectra (MS) were recorded by MALDI-TOF (time-of-flight) on a 4800 plus MALDI-TOF/TOF analyzer (Applied Biosystems) in the positive ion mode with delayed extraction. Mass spectra were recorded with an acceleration voltage of 20 kV. The intense peak was subjected to MS/MS. Collision-induced dissociation was performed at a collision energy of 1 kV with collision gas pressure of 32 ∼1 × 10−6 Torr. The resulting MS/MS spectrum was annotated manually assisted by GPMAW (Lighthouse data, Odense, Denmark). Four biological replicates were analysed.

**References:**

1 Rappsilber, J., Ishihama, Y. & Mann, M. Stop and go extraction tips for matrix-assisted laser desorption/ionization, nanoelectrospray, and LC/MS sample pretreatment in proteomics. *Anal. Chem.* **75**, 663-670 (2003).

2 Zagrobelny, M. *et al.* 454 pyrosequencing based transcriptome analysis of *Zygaena filipendulae* with focus on genes involved in biosynthesis of cyanogenic glucosides. *BMC Genomics* **10**, 574 (2009).
